# Endonucleolytic RNA cleavage drives changes in gene expression during the innate immune response

**DOI:** 10.1016/j.celrep.2024.114287

**Published:** 2024-05-31

**Authors:** Agnes Karasik, Hernan A. Lorenzi, Andrew V. DePass, Nicholas R. Guydosh

**Affiliations:** 1 Laboratory of Biochemistry and Genetics, National Institute of Diabetes and Digestive and Kidney Diseases, National Institutes of Health, Bethesda, MD 20892, USA; 2 TriLab Bioinformatics Group, National Institute of Diabetes and Digestive and Kidney Diseases, National Institutes of Health, Bethesda, MD 20892, USA; 3 Lead contact

## Abstract

Viral infection triggers several double-stranded RNA (dsRNA) sensors that lead to changes in gene expression in the cell. One of these sensors activates an endonuclease, ribonuclease L (RNase L), that cleaves single-stranded RNA. However, how the resultant widespread RNA fragmentation affects gene expression is not fully understood. Here, we show that this fragmentation induces the ribotoxic stress response via ZAKα, potentially through stalled ribosomes and/or ribosome collisions. The p38 and JNK pathways that are activated as part of this response promote outcomes that inhibit the virus, such as programmed cell death. We also show that RNase L limits the translation of stress-responsive genes. Intriguingly, we found that the activity of the generic endonuclease, RNase A, recapitulates many of the same molecular phenotypes as activated RNase L, demonstrating how widespread RNA cleavage can evoke an antiviral program.

## In brief

Karasik et al. uncovered that widespread mRNA degradation by RNase L leads to changes in gene expression and signaling. In particular, the authors found that RNA fragmentation promotes activation of the ZAKα signaling pathway. The findings establish how mRNA decay can promote innate immunity.

## Graphical Abstract

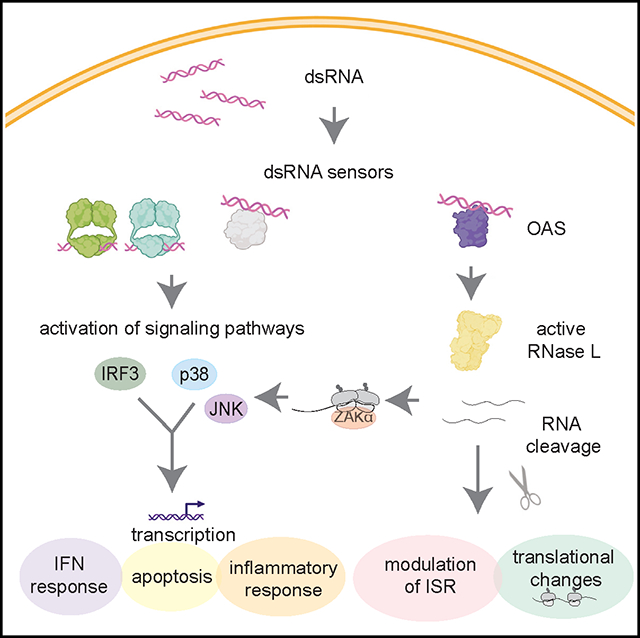

## INTRODUCTION

Foreign nucleic acid sensing is one of the major ways viral pathogens are recognized.^1^ Viruses either have an RNA genome or produce RNA intermediates, and thus, sensing of viral RNA in the cytoplasm is vital for recognizing and fighting against viruses. There are several double-stranded RNA (dsRNA) sensors that can recognize these viral RNAs.^1^ In particular, the interferon (IFN)-induced family of oligoadenylate synthetases (OAS1–3) produces an oligomer of adenylates chained together through 2′–5′ linkages (2-5A) upon dsRNA binding. 2-5A can selectively bind ribonuclease L (RNase L) to induce dimerization that is required for its transition from a latent to an activated state. Activated RNase L then cleaves a variety of host and viral RNAs, triggering many downstream processes and physiological changes in the cell. These changes include the loss of most mRNAs (and therefore gene expression in the cell), production of chemokines and cytokines, activation of inflammasomes, inhibition of cell migration, autophagy, senescence, and cell death.^2–5^ Intriguingly, the function of RNase L appears to extend beyond its role in protecting against viruses. OAS and/or RNase L mutations increase the risk for multiple types of cancer, including prostate and breast cancer,^6–9^ and autoimmune diseases.^10^

Another dsRNA sensor, protein kinase R (PKR), can induce a reduction in protein synthesis in the cell through phosphorylation of eukaryotic initiation factor 2α (eIF2α). Phospho-eIF2α inhibits initiation of translation of the majority of mRNAs to limit viral protein production and triggers a number of changes in the cell that are collectively referred to as the integrated stress response (ISR).^11–13^ In addition, the dsRNA receptors RIG-I and MDA5 induce the IFN and inflammatory responses by activating signaling that leads to transcription. Activation of the different dsRNA response pathways in the cytoplasm can have intersecting functions, whereby activated RIG-I induces gene expression, while activated RNase L and PKR reduce gene expression. However, how the different dsRNA sensing pathways, particularly RNase L, act together to achieve a strong antiviral response is largely unknown.

Activation of RNase L can induce and alter transcription.^14–17^ Importantly, more recent RNA-sequencing (RNA-seq) studies found that most transcripts are degraded (~60% to >99%) by RNase L. Interestingly, immune transcripts, such as IFN-β, are able to compensate for this degradation via transcriptional upregulation or resistance to cleavage due to underrepresentation of favorable RNase L cleavage motifs (UU or UA).^16,17^ Furthermore, many IFN-stimulated genes (ISGs) are upregulated during the dsRNA response in both wild-type (WT) and *RNASEL*-knockout (KO) cells, suggesting that they do not depend on RNase L, whereas upregulation of cytokines, such as *CXCL8* and *CXCL2*, and other immune genes, including *IFNL1* and 2 and *IFIT3*, was at least partially dependent on RNase L.^17^ One limitation of the existing studies that used RNA-seq is that RNase L was activated by a dsRNA mimic (poly(I:C)), an approach that simultaneously activates many other pathways.^16,17^ These pathways can mask RNase-L-specific effects and make it difficult to assess how RNase L activation and the other dsRNA pathways act together to induce the antiviral immune response.

Importantly, activation of RNase L can also induce pathways related to the ribotoxic stress response (RSR),^18,19^ which triggers apoptosis through transcription activated by JNK (c-JUN N-terminal kinase) signaling. JNK is a mitogen-activated protein kinase (MAPK) family member. While several MAP2K family members can phosphorylate JNK,^19^ the mechanism of how they are activated by RNase L has remained unclear. The RSR was originally shown to be activated by defective translation, so it was suggested that pathways related to the RSR were induced when RNase L damaged ribosomes by cleaving rRNA.^18–20^ However, more recently, it was revealed that ribosomes with cleaved rRNAs are functional, suggesting that ribosome damage may not be responsible for activating the RSR.^16^ In addition, the RSR was recently shown to include activation of the MAP3K family member ZAKα, which senses collisions between ribosomes and phosphorylates MAP2Ks that, in turn, phosphorylate JNK and another MAPK, p38.^21–24^ These observations suggest that activation of ZAKα by stalled ribosomes and/or potentially by ribosome collisions could occur when RNase L induces the RSR.

More broadly, the overall loss of translated mRNA in the cell upon RNase L activation is expected to change the balance of free ribosomal subunits and mRNAs.^16^ This altered balance could modulate the translation efficiency of the remaining full-length transcripts. Consistent with this, we previously observed an increase in the relative proportion of ribosomes in alternative open reading frames (ORFs), or “altORFs,” following RNase L activation.^25^ We proposed that translation of these altORFs became more favorable as excess free ribosomes promoted translation initiation at the 5′ ends of the RNA fragments created by RNase L. However, how RNase L and other dsRNA pathways lead to changes in translation on mRNAs remained unclear.

Here, we investigate how direct activation of RNase L, and attendant global mRNA degradation, contributes to the activation of the innate immune response. We found that RNase-L-mediated RNA cleavage contributed to multiple parts of the antiviral transcriptional response and, in particular, induced the ribosome stalling and collision sensor ZAKα. In addition, activation of RNase L also changed how efficiently intact mRNAs were translated, in some cases by inhibiting the effect of eIF2α phosphorylation. Our results highlight the multifaceted role of RNase L and endonucleolytic cleavage in promoting antiviral functions.

## RESULTS

### Activation of RNase L induces transcription and phosphorylation of ZAKα

**Figure 1.**
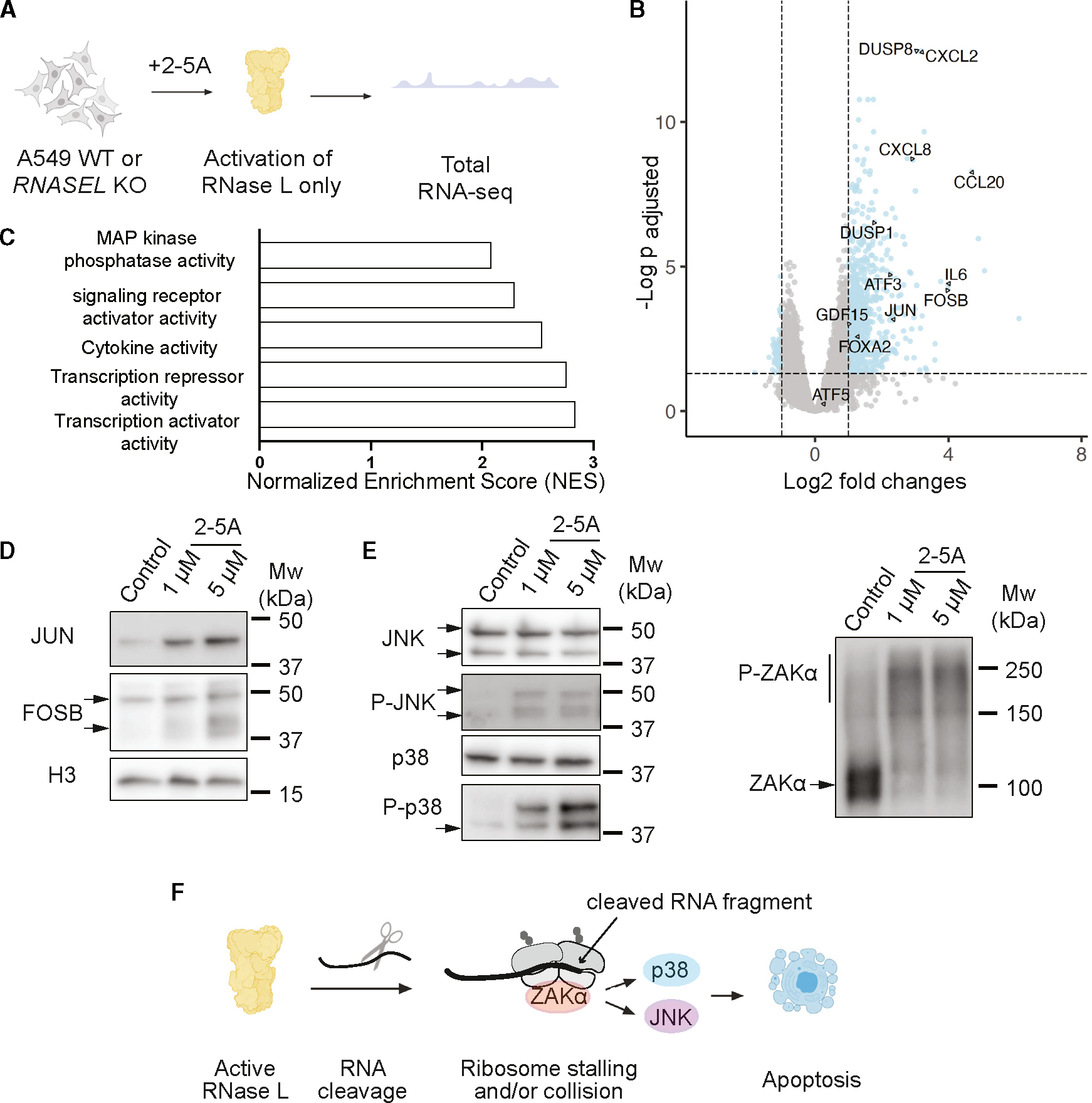
RNase L activation leads to transcriptional changes through ZAK, JNK, and p38 (A) Schematic of RNA-seq experiments (*n* = 5 experiments). (B) Volcano plot showing upregulation of example cytokines and transcription factors during 2-5A treatment. Adjusted *p* values were calculated by the Benjamini-Hochberg method using DESeq2. (C) GO analysis shows that upregulated genes are enriched in genes related to transcription activities. (D) Western blots show that transcription factors FOSB (isoforms are marked with black arrows) and JUN are increased by 2-5A activation (*n* = 4). (E) Western blots show that JNK (isoforms marked with black arrows, *n* = 5), p38 (*n* = 7), and ZAKα (*n* = 3) are activated in A549 cells after 2-5A treatment. (F) Working model of JNK/p38 pathway activation by RNase L. RNase L cleaves translated mRNAs. At the end of the resulting fragments, ribosomes collide and activate ZAKα and subsequently JNK and p38. See also Figures S1 and S2 and Table S1.

To measure changes in the transcriptome under activation of RNase L, but not other components of the dsRNA-induced innate immune response, we performed RNA-seq on WT and *RNASEL*-KO cells that were transfected with 2-5A (1 μM), the direct and specific activator of RNase L (Figure 1A). Since RNase L cleaves rRNA, we assayed rRNA degradation to assess the level of RNase L activation.^26^ Across all replicates, we found that rRNA degradation was similar (within a 2-fold difference, Figure S1A). Based on previous reports that used spike-in oligos for normalization,^16,17^ these levels of rRNA degradation correspond to a loss of ~60% to >99% of individual mRNAs in the cell. We therefore assume that the absolute abundance of most mRNAs in the treated cells under study here substantially decreases (“baseline loss”).

Then, we analyzed differential expression patterns in 2-5A-treated vs. untreated cells using DESeq2.^27^ The differential expression changes in our experiments represent differences in relative abundance of the small pool of mRNA that is not degraded. We infer that mRNAs that appear upregulated (described throughout as relative “upregulation”) actually decrease in abundance, but to a lesser extent than the general population, due to resistance to RNase L cleavage activity or compensation by active transcription. The differential expression values therefore characterize how the global loss of mRNA varies across the transcriptome. We found hundreds of differentially expressed genes (DE genes; *p*_adjusted_ < 0.05) in 2-5A-treated WT cells, revealing that the level of these mRNAs significantly differs from the baseline loss (Figure 1B). Treatment with 2-5A did not induce many changes in differential gene expression in *RNASEL*-KO cells (Table S1), as expected, since 2-5A is specific for RNase L. DE genes that appeared to be upregulated by RNase L activation (Figure 1B), defined as log2 fold changes >1, included transcripts that encode proinflammatory proteins and cytokines (e.g., *CXCL2*, *CCL20*, *CXCL8*, and *IL6*), suggesting that direct RNase L activation stimulates an inflammatory response. In contrast, we did not detect an IFN response, such as increased transcript levels for *IFNB1*. These findings are largely consistent with previous RNA-seq data from human cells (WT vs. *RNASEL* KO) that were transfected with poly(I:C)^16,17^ and were consistent across replicates (Figure S1B).

We noted that some of the genes that appear to be upregulated in 2-5A-treated cells encode transcription factors or activators, such as *JUN*, *FOSB*, *FOXA2*, *GDF15*, and *ATF3* (Figure 1B). This is consistent with previous findings for some of these genes, such as *FOXA2* and *GDF15*.^14,28^ Gene Ontology (GO) analysis of molecular functions offered additional support, since several of the top categories of DE genes were related to transcription regulation (Figure 1C). This finding suggests that the apparent increase in some transcript levels in our differential expression analysis could be due to new transcription that is induced by these factors. Since RNase L reduces global mRNA levels in the cell, it is unclear whether the relative upregulation of these transcription factor genes observed in our RNA-seq data is sufficient to increase production of protein. Interestingly, we found that protein levels for FOSB and JUN increased in 2-5A-treated WT, but not *RNASEL*-KO, cells using western blotting (Figures 1D and S2A). This finding suggests that activation of RNase L can actually raise the abundance of these proteins, despite heavy baseline loss of mRNA, leading to downstream transcriptional activation.

Next, we assessed which pathways are triggered in 2-5A-treated cells that could explain the apparent transcriptional upregulation. We found that several target genes in the JNK, p38, ERK, and NF-κB pathways appeared to be increased in our data, suggesting that at least some of these pathways (which are known to have overlapping targets) are activated. To dissect which of these pathways are specifically responsible for the observed transcriptional changes in our dataset, we used western blotting to assess activation through phosphorylation of these pathways. We found that RNase L can activate JNK and p38 (Figures 1E and S2B), but we did not detect activation of pathways related to ERK (Figure S2C, ERK phosphorylation) or NF-κB (Figure S3, p65 nuclear fraction). These results suggest that transcriptional changes observed in the cells here are driven, in part, by the activation of JNK and p38. We also noticed that multiple *DUSP* mRNAs, such as *DUSP1* and 8, which encode negative regulators of the JNK pathway, were upregulated (Figures 1B and S1B), suggesting a need for modulation of JNK when RNase L is activated. Interestingly, JNK activation can be apoptotic or induce autophagy. Here, we observed the induction of proapoptotic genes, such as *JUN*, but not genes expressed during autophagy, such as ATF5.^29^

It has been suggested that activation of RNase L can trigger the RSR, which can lead to downstream activation of the JNK and p38 pathways and apoptosis.^19,21^ Since it was demonstrated that this can involve the ribosome stalling and collision sensor and kinase ZAKα, we tested whether ZAKα is activated in 2-5A-treated cells by examining its phosphorylation state by western blotting. We found that ZAKα is strongly activated by 2-5A treatment in WT but not in *RNASEL-KO* cells (Figures 1E and S2B, Phos-Tag gel shows shift from hyperphosphorylation), and these findings were consistent in another cell line, MCF10A (Figure S2D). We further confirmed that p38 and JNK MAPKs are activated via ZAKα using *ZAK*-KO MCF10A cells (Figure S2D). We also found that activation of p38 and JNK was dependent on the catalytic activity of RNase L (Figure S2E), suggesting that generation of RNA fragments is needed for triggering these pathways. We also observed signatures of apoptosis, such as blebbing, and elevated levels of pro-caspase-3 cleavage during 2-5A treatment in WT but not in *ZAK*-KO cells (Figures S2F and S2G). This further indicates that RNase L promotes apoptosis via ZAKα and the RSR.

Our data are consistent with a model in which RNase L activation causes ribosome stalling and/or collisions that, in turn, cause phosphorylation of ZAKα. One possible source of stalled ribosomes was suggested by our previous observation that activation of RNase L leads to translation of fragmented mRNAs.^25^ Ribosomes that translate mRNA fragments could reach 3′ ends without terminating (Figure 1F), an event known to lead to the formation of ribosome collisions.^30^

### The OAS-RNase L pathway acts together with other dsRNA-sensing pathways to achieve strong activation of innate immune signaling

**Figure 2.**
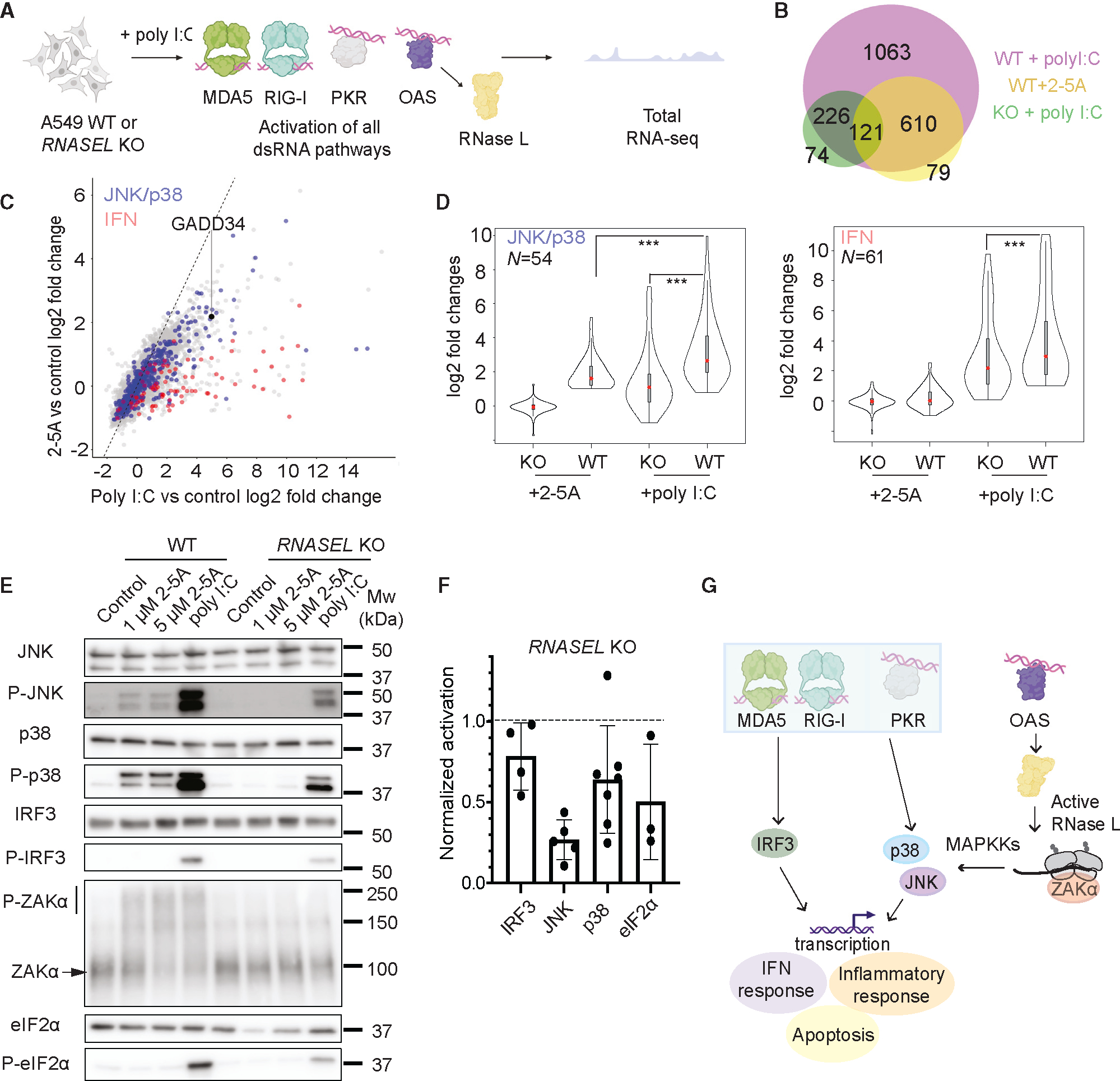
OAS-RNase L and other dsRNA pathways act together to induce the proinflammatory response (A) Schematic of RNA-seq experiments (*n* = 3–5 experiments). (B) Venn diagram showing the overlap of DE genes in poly(I:C)-treated WT (magenta), 2-5A-treated WT (yellow), and poly(I:C)-treated *RNASEL-KO* cells (green, abbreviated KO). (C) Correlation between differential expression changes in 2-5A- (RNase L alone) vs. poly(I:C)-treated cells. Blue and red dots represent JNK/p38 MAPK activation-related or IFN-stimulated genes, respectively. Black dot represents *GADD34*. (D) Comparison of JNK/p38 and IFN pathways under 2-5A (RNase L alone) vs. poly(I:C) activation. *p* < 0.001, indicated by three asterisks and calculated by using Student’s t test. (E) Effects of activating the OAS-RNase L pathway on phosphorylation of JNK (*n* = 5), p38 (*n* = 7), ZAKα (*n* = 3), eIF2α (*n* = 3), and IRF3 (*n* = 4) assayed by western blotting. (F) Quantification of blots in (E). Activation levels were normalized to that of poly(I:C)-treated WT cells. Bars represent standard error. (G) Model for RNase L acting synergistically with the other dsRNA pathways to enhance the antiviral effect for JNK and p38 via ZAKα. See also Figure S3 and Table S1.

We next asked whether activation of RNase L modulates the effects of other pathways that are typically activated during infection by other dsRNA sensors.^31,32^ To simultaneously activate multiple pathways and test RNase L’s role, we transfected WT and *RNASEL-KO* A549 cells with poly(I:C), a dsRNA mimic (0.25 μg/mL), and performed RNA-seq (Figure 2A). Poly(I:C) is known to strongly activate OAS and, in turn, RNase L, as well as other dsRNA-sensing pathways, including PKR and RIG-I/MDA5. First, we assessed the levels of RNase L activation by measuring the level of rRNA degradation and comparing it to that in cells treated by the direct activator, 2-5A. We found that in all replicates of poly(I:C)- and 2-5A-treated cells, the level of rRNA degradation was comparable (within 2-fold), suggesting similar levels of RNase L activation (Figure S1A).

Next, we determined the DE genes in WT cells upon poly(I:C) treatment. We found that poly(I:C) treatment resulted in a higher number of relatively upregulated DE genes (~2,000, log2 fold changes >1, *p*_adjusted_ < 0.05) compared to 2-5A treatment (~800 genes) and that additional pathways were activated, as expected given the broader effects of poly(I:C) (Figure 2B). In particular, in both WT and *RNASEL-KO* cells, the PKR and RIG-I/MDA5 pathways were activated, as judged by increased levels of eIF2α phosphorylation (shown previously in Karasik et al.^25^ and discussed further below) and the increase in ISGs (Figure 2C; Table S1), respectively. Consistent with this, we observed that *PPP1R15A* (*GADD34*), a gene involved in the ISR (see below) and known to be transcribed as a result of both IFN production via the IRF3/7 pathway and PKR activation,^33^ also exhibited a relative increase compared to other transcripts (~32-fold) in poly(I:C)-treated WT cells (Table S1; Figure 2C).

Notably, we also found that most genes that are DE upon 2-5A treatment were also DE in poly(I:C)-treated cells (Figure 2B, note overlap between yellow- and magenta-colored circles, Pearson’s R^2^ = 0.92). This suggests that these changes are largely unimpaired in cells treated with poly(I:C) and are not masked by the effects of the other activated dsRNA pathways. We noted that many genes that did not meet the criteria for significant differential expression in WT cells treated with 2-5A changed in ways that closely mirrored changes observed in cells treated with poly(I:C) (Pearson’s R^2^ = 0.69 for DE genes in this subset). This correlation indicates that the small relative gene expression changes in 2-5A-treated cells that do not meet the differential expression threshold may indicate partial activation of pathways that are amplified when other dsRNA sensors are triggered in poly(I:C)-treated cells.

To more closely investigate how simultaneous activation of RNase L and other pathways triggered by dsRNA affects the JNK and p38 pathways, we examined the mRNA levels of target genes and phosphorylation of the proteins themselves. Based on our finding above that 2-5A treatment (RNase L activation alone) activates the JNK and p38 pathways (Figures 1E and 2C, blue dots), we defined a subset of 54 transcripts that are commonly associated with JNK and p38 and are activated in 2-5A-treated samples (see STAR Methods for more details). Since ~50% of the genes downstream of JNK and p38 overlap, we analyzed these MAPK pathways together. We assessed transcriptional induction of JNK/p38 downstream targets under activation of RNase L only (WT + 2-5A), other dsRNA pathways only (*RNASEL-KO* cells + poly(I:C)), or both pathways (WT + poly(I:C)) (Figure 2D). As expected based on the selection of targets, we found that these JNK/p38-related mRNAs were highly induced in WT + 2-5A-treated cells. We also noted some activation due to other dsRNA pathways alone (*RNASEL-KO* + poly(I:C)). Interestingly, when combined, the RNase L and other dsRNA pathways together suggested the effects may be additive, since the level of induction was highest in WT cells treated with poly(I:C) (Figure 2D). We therefore asked whether these trends were supported by the levels of JNK and p38 phosphorylation, as measured by western blotting (Figure 2E). In support of additivity between the RNase L and the other dsRNA pathways, we observed the strongest phosphorylation of JNK and p38 when both RNase L and the other dsRNA pathways were triggered compared with either one alone (Figures 2E and 2F).

In addition, we found that ZAKα, which we showed to be phosphorylated by 2-5A treatment (Figure 1E), was also phosphorylated in poly(I:C)-treated WT cells to a similar extent, suggesting that activation of ZAKα by RNase L is not affected by the other dsRNA pathways (Figure 2E). Importantly, ZAKα phosphorylation was not detectable in *RNASEL-KO* cells that were treated with poly(I:C), suggesting its activation requires RNase L activity. This model therefore implies that JNK and p38 phosphorylation in the absence of RNase L (Figure 2E) must use a MAP3K other than ZAKα and that, together with ZAKα, affords the strongest induction of p38/JNK targets in cells treated with poly(I:C) (Figure 1E).

Activation of ZAKα by ribosome stalling and/or collisions can simultaneously occur with activation of the GCN2 kinase, which phosphorylates eIF2α and is also activated by ribosome collisions.^21,23^ Hence, we wondered if RNase L could contribute to eIF2α phosphorylation through ribosome collisions that activate GCN2. We did not detect eIF2α phosphorylation after 2-5A treatment, consistent with previous reports.^5,25^ However, we found that eIF2α phosphorylation levels were substantially decreased in poly(I:C)-treated *RNASEL-KO* cells compared to WT cells (Figures 2E and 2F), which is also consistent with previous reports.^17^ This finding shows that activation of RNase L increases eIF2α phosphorylation under some conditions, perhaps by enhancing PKR activation, activating another kinase such as GCN2, and/or reducing the activity of an eIF2α phosphatase.

Next, we assessed if activation of RNase L affects the IFN response that is triggered by dsRNA binding to MDA5 and RIG-I (Figure 2C, red dots). We defined a subset of ISGs (*N* = 61, see STAR Methods for details) that were upregulated in WT cells treated with poly(I:C) (Figure 2D, right). We found no activation of these genes with 2-5A treatment, consistent with previous RNA-seq findings in A549 cells.^16,17,25^ In cells treated with poly(I:C), we observed some decrease in activation for this limited subset of genes in *RNASEL-KO* cells compared to WT cells (Figure 2D). We also visualized the phosphorylation of IRF3 by western blotting, one of the main transcription factors that induces ISGs in response to IFN (Figures 2E and 2F). We were unable to detect activation of IRF3 in cells treated with 2-5A but observed activation in cells treated with poly(I:C), in agreement with our RNA-seq analysis. We also observed a small decrease (~20%) in activation of IRF3 in *RNASEL-KO* cells treated with poly(I:C), in line with our observations from RNA-seq and a previous report.^17^ These findings suggest that RNase L may have a minor role in augmenting activation of other dsRNA sensors in these cells.^31,32^

We also asked whether RNase L can contribute to the other pathways that play a role in the dsRNA-induced innate immune response, such as NF-κB and ERK. Activation of NF-κB (p65) was only detectable in poly(I:C)-treated cells, and the levels of activation appeared to be similar in both WT and *RNASEL*-KO cells, indicating that RNase L has no further effects on this pathway (Figure S3). We also found that 2-5A or poly(I:C) treatment had no effect on ERK activation in A549 cells, suggesting that the observed effects in our experiments are not due to activation of ERK (Figure S3).

Taken together, our analysis shows that activated RNase L can act together with other dsRNA pathways to promote p38 and JNK signaling but has only minor effects on the activation of IRF3 (Figure 2G).

### A generic endonuclease, RNase A, induces transcriptional changes similar to those triggered by RNase L

**Figure 3.**
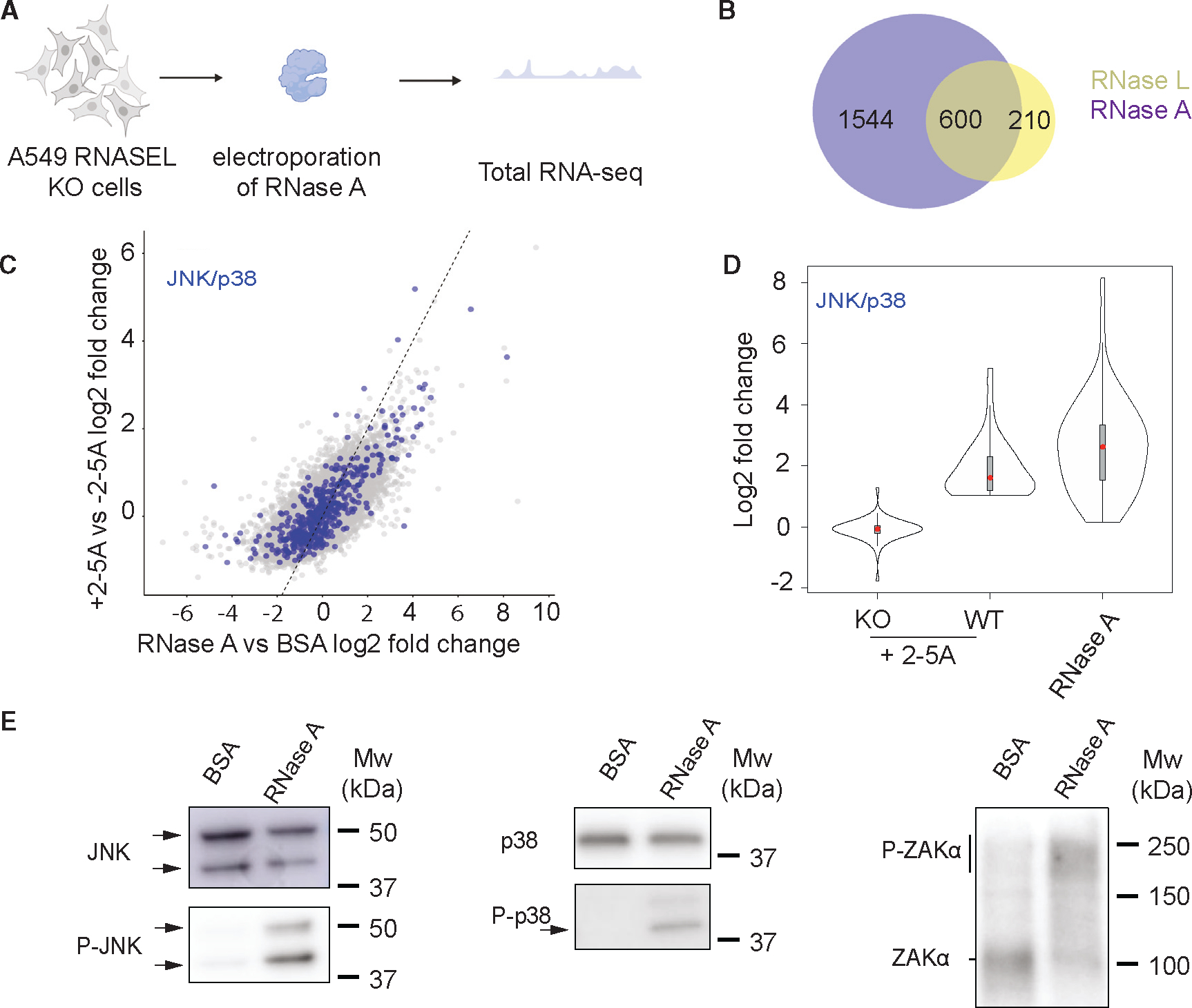
Global RNA cleavage in the cell leads to transcriptional changes (A) Schematic of RNA-seq experiments (*n* = 2 experiments). (B) Venn diagram showing the number of upregulated genes in RNase-A-electroporated samples (purple) and 2-5A-treated cells (yellow). (C) Comparison of RNA log2 fold changes between RNase-L-activated vs. RNase-A-electroporated samples. Blue dots represent transcripts related to JNK and p38 activation. (D) Comparison of JNK/p38 pathways with RNase L vs. RNase A activity. (E) JNK, p38, and ZAKα are activated by RNase A treatment, as shown by western blots (*n* = 3). Different JNK isoforms are marked with black arrows. See also Figure S4 and Table S1.

Next, we wanted to assess what changes directly result from the global RNA cleavage that occurs after RNase L activation. RNase L is known to interact with several proteins, including those that make up the cytoskeleton and the nucleopore complex.^34–39^ Thus, it is possible that some of the observed changes upon RNase L activation and dimerization arise from functional protein-protein interactions and not RNA cleavage. To further investigate, we electroporated a generic RNase, RNase A (bovine pancreatic ribonuclease), into *RNASEL*-KO A549 cells and performed RNA-seq after 4.5 h (Figure 3A). RNase A is a small endonuclease that primarily cleaves at pyrimidine bases (U and C) in single-stranded RNAs (ssRNAs).^40,41^ As a control, we electroporated similar amounts of bovine serum albumin (BSA), since it is similar in size, but has no RNase activity. We anticipated that RNase A would cause widespread RNA decay but would lack RNase-L-specific interactions. We followed RNase A activity by monitoring rRNA cleavage, similar to our above experiments with RNase L. We observed that rRNAs are degraded in RNase-A-electroporated cells, but with different RNA cleavage patterns (Figure S4A).

We found >2,000 DE genes in RNase-A-electroporated cells (Table S1). As noted for RNase L activation above, gene expression is reported in relative terms above or below the average global loss of mRNA; therefore, genes that appear to be upregulated may simply be less downregulated than those in the same treatment group. Interestingly, most upregulated DE genes found in 2-5A-treated cells overlap with those identified here for RNase A activation (~74% of those in the RNase L dataset, Figures 3B, 3C, and S4B), and the expression levels showed correlation (Pearson’s R^2^ = 0.53, Figure 3C). This suggests that most RNase-L-dependent differential expression changes are the direct result of RNA cleavage rather than potential indirect non-enzymatic activities.

Next, we assessed if targets of the JNK signaling pathway were upregulated in RNase-A-electroporated cells, as they were in 2-5A-treated ones. We found that the JNK- and p38-pathway-related mRNAs were upregulated in RNase-A-electroporated samples (Figure 3D). JNK and p38 phosphorylation was also confirmed by western blotting (Figure 3E). Strikingly, we found that ZAKα was also phosphorylated (Figure 3E). Our results therefore indicate that transcriptional changes, particularly through the JNK and p38 pathways, can be induced by non-specific RNA cleavage, regardless of the effector endonuclease.

### RNA fragmentation by generic RNases induces altORF translation

**Figure 4.**
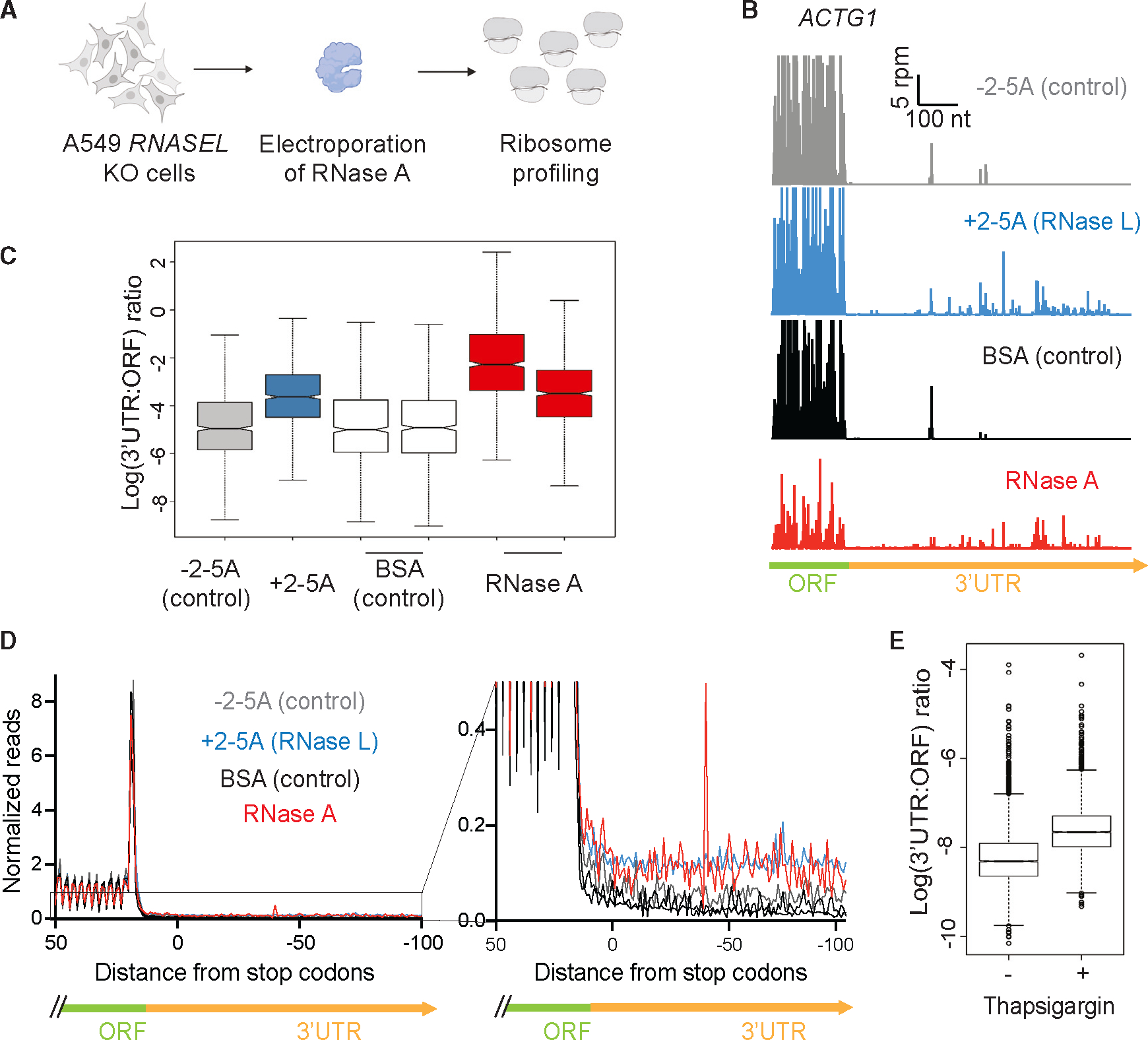
RNA fragmentation leads to altORF translation (A) Schematic of ribosome profiling experiments (*n* = 2 experiments). (B) Ribosome profiling reads in the 3′ UTR of *ACTG1* show the signature of altORF translation in RNase-A-electroporated and RNase-L-activated A549 cells. (C) Increased 3′ UTR:ORF ratios indicate higher fraction of translation in 3′ UTRs when active RNases are present in the cell. (D) Metagene plot around the stop codon of main ORFs (left) reveals increased proportion of ribosome footprints in 3′ UTRs (right, zoom-in). (E) Increased 3′ UTR:ORF ratios indicate altORF translation during thapsigargin treatment (UPR activation), but not in untreated cells. In boxplots of UTR:ORF density ratios (C and E), boxes represent the interquartile range (IQR) and horizontal lines are the median. Whiskers show 1.5 * IQR and notches give $1.58\;*IQR/\sqrt{N}$. In (B), (C), and (D), data shown for RNase L activation (+2-5A) were obtained from Karasik et al.^25^ See also Figure S5.

Previously, we examined the translation that takes place in RNase-L-activated cells by using ribosome profiling to find where ribosomes translate the reduced pool of mRNAs.^25^ One of the most striking findings from our previous study is that the relative abundance of ribosome footprints increased in non-coding regions of transcripts, including the 5′ and 3′ UTRs, as well as out-of-frame parts of coding sequences. This likely results when ribosomes initiate translation at the first available ORF on the 3′ mRNA fragments that are generated by RNase L, a phenomenon that we termed “altORF translation.”^25^ The model predicts that the activity of any generic endonuclease should lead to the same outcome. To test this hypothesis, we electroporated RNase A into *RNASEL*-KO A549 cells, as described above, and performed ribosome profiling on those same samples after 4.5 h (Figure 4A).

The major hallmarks of altORF translation that we previously observed in RNase-L-activated cells were recapitulated in the cells into which RNase A was electroporated (Figures 4B–4D). The most striking signature of altORF translation is a relative increase in ribosome footprints in the 3′ UTR. Cells in which RNase A was active exhibited similar ribosome profiling patterns in the 3′ UTRs, consistent with the altORF translation model (Figure 4B, *ACTG1* as an example gene model). To assess characteristics of altORF translation globally, we computed the ratios of ribosome profiling footprint levels in 3′ UTRs and the main ORFs of mRNAs (3′ UTR:ORF ratios), as we did before for active RNase L. We found that in all RNase-treated samples, 3′ UTR:ORF ratios increased compared to respective controls (Figure 4C).^25^ Increased 5′ UTR:ORF ratios were also observed in RNase-L-activated cells,^25^ and we found similar patterns when computing 5′ UTR:ORF ratios for RNase-A-treated cells (Figure S5A). Increased reads in the 5′ and 3′ UTRs were also observed in metagene averages at the start and stop codons of main ORFs for RNase-A-electroporated samples compared to the control (Figures 4D and S5B), further confirming increased relative ribosome occupancy and potential altORF translation in the UTRs.

The data from cells that were electroporated with RNase A suggest that the phenomenon of altORF translation takes place whenever any generic endonuclease, not only RNase L, is active in the cytoplasm. Electroporation of RNase A is artificial, and we wondered whether another naturally activated endonuclease would trigger the translation of altORFs. The endonuclease IRE1 is homologous to RNase L and plays a pivotal role in the unfolded protein response (UPR), since it is activated by protein misfolding in the ER.^42^ IRE1 can cleave somewhat generically at UGC sequence motifs, leading to regulated IRE1-dependent decay (RIDD) and potentially to altORF translation.^30,43^ To test this hypothesis, we analyzed a publicly available ribosome profiling dataset where human cells were treated with thapsigargin, a potent inducer of the UPR and IRE1^44^ (GEO: GSE103719). We found that thapsigargin-treated cells also exhibited characteristics of altORF translation, including increased 3′ UTR:ORF ratios (Figure 4E). These results show that RNase L is not the only naturally activated endonuclease that can lead to the translation of altORFs.

### RNA fragmentation can modulate translational efficiency

**Figure 5.**
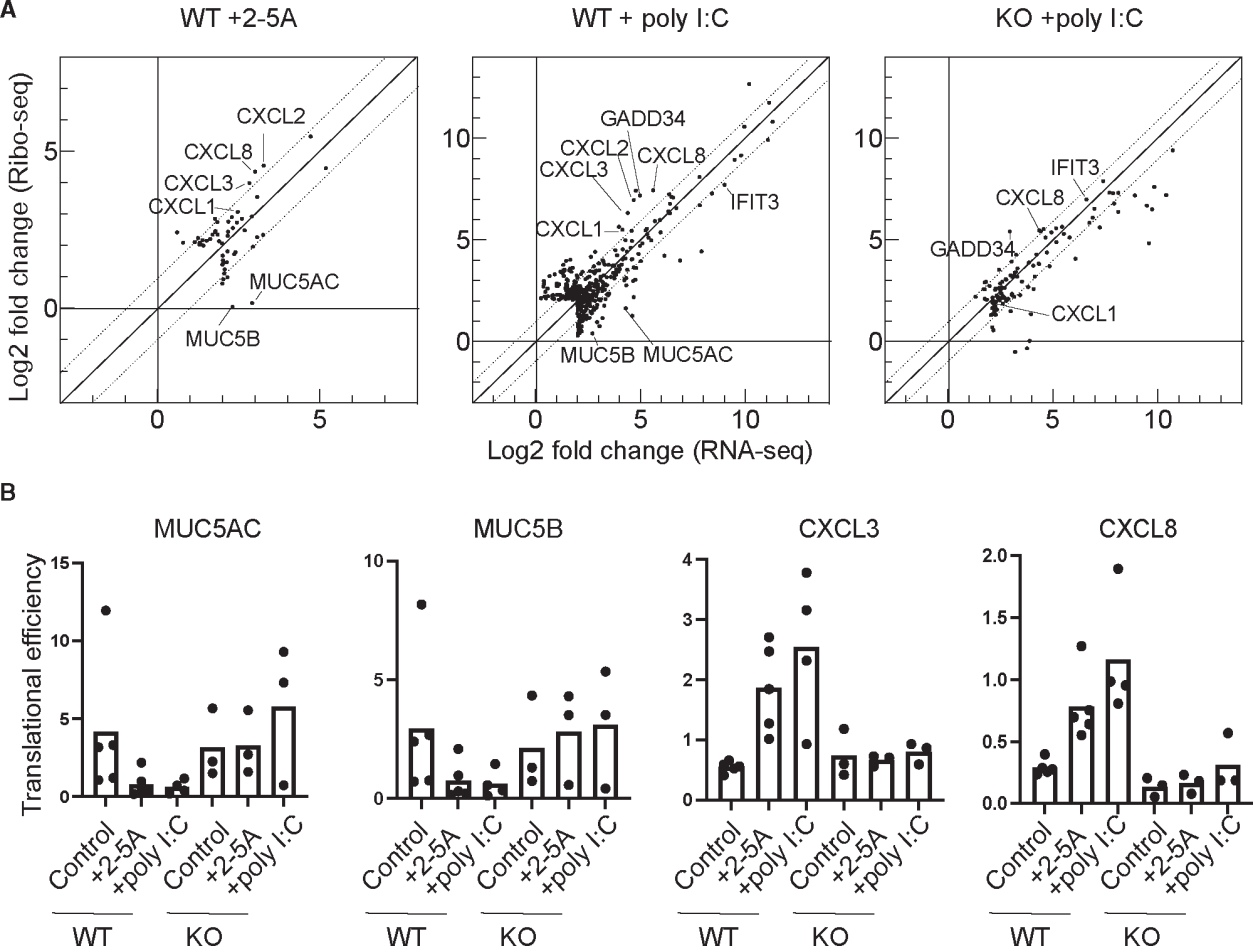
RNase L activation influences translational control and effects of poly(I:C) treatment (A) Comparison of changes in RNA-seq vs. ribosome profiling when RNase L only is activated (left), RNase L and other dsRNA sensors are activated (center), orother dsRNA sensors only are activated (right) (*n* = 3–5). Dotted diagonal lines represent TE changes of >2-fold. (B) TEs from replicates of genes of interest show consistency. *RNASEL* KO is abbreviated as KO in (A) and (B) (*n* = 3–5). See also Figures S6 and S7 and Table S2.

The major effect on gene expression of active RNase L is that the majority of mRNAs are lost, resulting in reduced protein synthesis for most genes. However, transcriptional upregulation counteracts this and, in some cases, overrides (Figure 1D) this effect. In addition, it is conceivable that gene expression from the remaining mRNAs could be tuned at the translational level and thus further refine the amount of protein synthesized from the remaining transcriptome. To investigate whether RNase L activity alters which mRNAs are translated, we took advantage of our ribosome profiling data (reported previously^25^ and in additional datasets here) and, with its matched RNA-seq data (reported here), assessed the translational efficiency (TE) of coding sequences. The TE metric is defined as the ribosome footprint level in a coding region, normalized to the corresponding RNA-seq level, and serves as a proxy for the relative loading of a transcript by ribosomes. We therefore plotted the change in ribosome footprint levels against the corresponding change in RNA-seq level (Figure 5A and Table S2) for genes where either of these values was found to change significantly by DESeq2 (see STAR Methods). Those genes with large changes in TE will appear farthest from the diagonal (slope >1 indicates TE increase; <1 TE decrease). We noted several cases where dots were shifted away from the diagonal, suggesting functionally important regulation of translation.

Some of the largest changes we observed affected a group of transcripts that were expressed at higher relative levels when RNase L was active at the mRNA level (via 2-5A or poly(I:C) in WT but not in *RNASEL*-KO cells) but were counter-balanced by downregulation of expression at the translational level (lower TE, Figure 5A, dots to the right of the origin and below the dotted diagonal). This category includes the extracellular matrix proteins *MUC5AC* and *MUC5B* (Figures 5A and 5B). In another class of genes that includes *CXCL3* and *CXCL8*, we observed that relative mRNA levels were increased upon RNase L activation (via 2-5A or poly(I:C) in WT but not in *RNASEL*-KO cells) and that the level of ribosome footprints also increased to a larger extent and would therefore be expected to augment gene expression (Figures 5A, dots to the right of the origin and above the dotted diagonal, and 5B).

**Figure 6.**
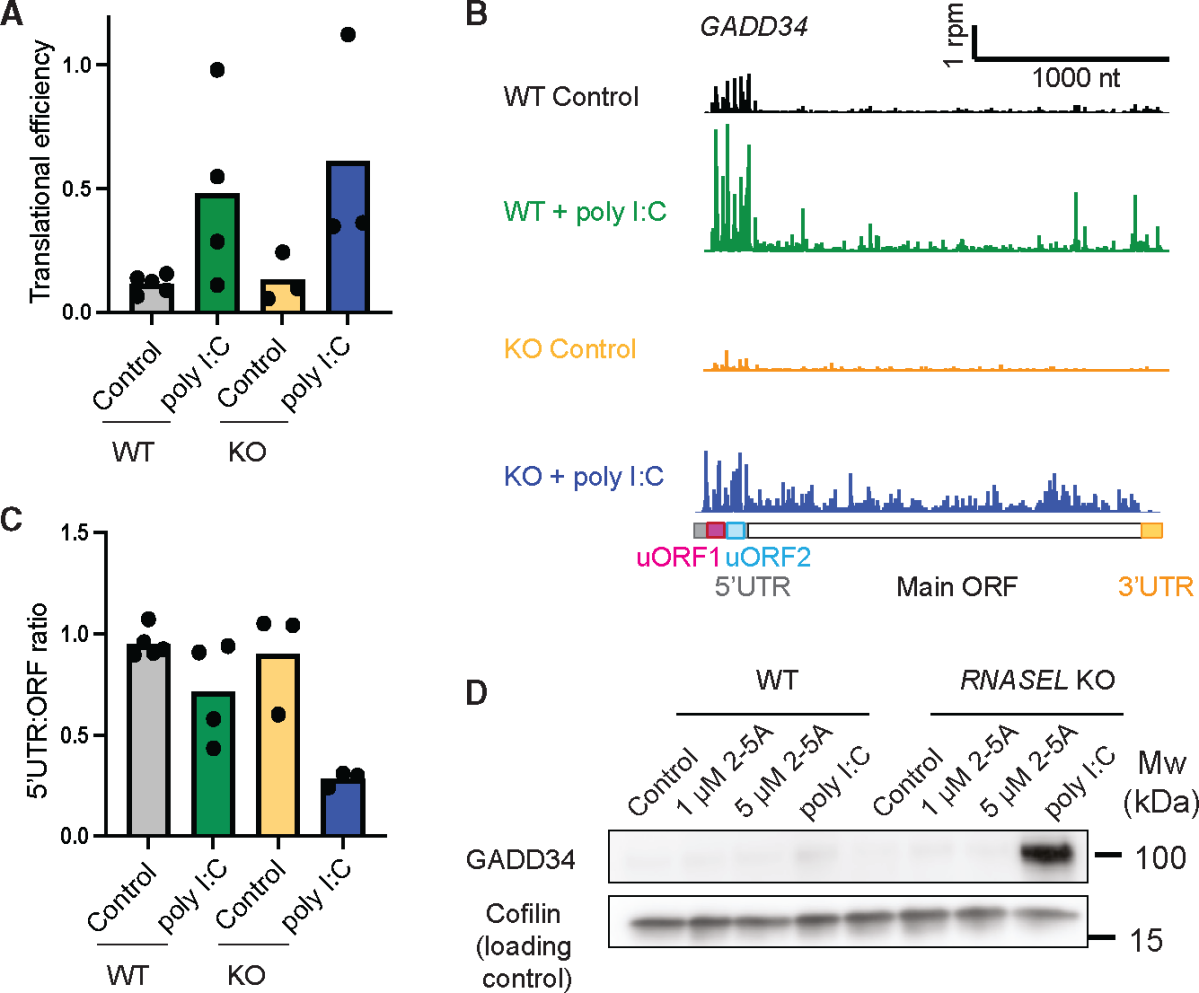
RNase L modulates elements of the integrated stress response (A) Individual replicate (and mean) TEs of *GADD34* mRNA (*n* = 3–5). (B) Ribosome profiling on *GADD34* (*PPP1R15A*) suggests a change in 5′ UTR vs. main ORF translation ratio in *RNASEL*-KO cells during the ISR compared to WT cells and their respective controls. (C) 5′ UTR:main ORF ratios of ribosome profiling data show that this value is more strongly reduced upon poly(I:C) treatment in cells where RNase L is absent. (D) Western blot for GADD34 shows increased levels of protein in poly(I:C)-treated *RNASEL*-KO cells, reflecting both the lack of mRNA degradation and the enhanced TE (*n* = 3).

Given the role of *GADD34* in the ISR, we further examined the ribosome profiling data to see how its translation is affected by RNase L. The ISR is activated when PKR binds dsRNA and phosphorylates the translation initiation factor eIF2α, ultimately resulting in a loss of its ability to promote translation initiation.^45^ Phosphorylation of eIF2α generally limits the loading of ribosomes on mRNAs in the cell, reducing their TE. However, there is a small class of mRNAs, including *ATF4*, *GADD34* (*PPP1R15A*), and *CHOP* (*DDIT3*), that undergo the opposite response and are translationally activated.^11^ The transcripts for *ATF4*, *CHOP*, and *GADD34* contain one or more ORFs in their 5′ UTR (uORFs) with unique features that allow them to suppress translation of the main ORF in non-stressed cells but activate it when eIF2α becomes phosphorylated.^11,46^ As noted above, the TE for *GADD34* was observed to increase in poly(I:C)-treated cells (WT or *RNASEL* KO) (Figure 6A), although less in WT than in *RNASEL*-KO cells, despite higher levels of eIF2α phosphorylation (Figure 2E). Several explanations could account for this. One possibility is that RNase L could directly affect TE by altering the shift in translation from the uORFs to the main ORF. To determine whether RNase L had an effect on uORF-based regulation, we quantified the ratio of ribosome footprints in the 5′ UTR and compared it to footprints that mapped to the main ORF (Figures 6B and 6C; see STAR Methods). We noted a substantial decrease in uORF translation relative to main ORF translation in poly(I:C)-treated WT or *RNASEL-KO* cells due to eIF2α phosphorylation, as expected.^46^ However, this decrease was less in the case of the WT cells compared to *RNASEL-KO* cells (Figures 6B and 6C), consistent with a role for RNase L activation in this shift. Similar RNase-L-dependent shifts in 5′ UTR vs. main ORF ribosome footprints were seen for *ATF4* and *CHOP* (Figures S6A and S6B). It has been shown that the *GADD34* mRNA is heavily targeted by RNase L, making the mRNA and protein product in poly(I:C)-treated *RNASEL*-KO cells much more detectable than in WT cells.^17^ Consistent with this, we also found that GADD34 protein levels increased in poly(I:C)-treated *RNASEL*-KO cells (Figure 6D). Thus, our observation that RNase L activity somewhat suppresses main ORF translation augments the underlying trend in expression that is driven by mRNA decay.

Since we observed that poly(I:C) induced higher translation efficiency in *RNASEL*-KO vs. WT cells for the gene *IFIT3* (Figure 5A, right), we more cosely examined the ribosome profiling data on IFIT proteins 1–3, which are stimulated by IFN and inhibit viral translation.^47^ Since these genes have low expression under conditions without poly(I:C) stimulation, we focused on data from cells treated with poly(I:C). We found that the 5′ UTRs of *IFIT1*, *2*, and *3* contained ribosome peaks at the start codons (non-canonical CUG or GUG instead of AUG) of potential uORFs (Figure S7A, green lines). These uORFs might inhibit main ORF translation, and it is conceivable that this inhibition could be regulated by eIF2α phosphorylation. This effect may be regulated by RNase L, since there was lower 5′ UTR translation relative to main ORF translation in poly(I:C)-treated *RNASEL*-KO vs. WT cells that was correlated with higher TE (Figures S7B and S7C). We also found that IFIT1 and IFIT2 protein levels increased in poly(I:C)-treated *RNASEL*-KO cells compared to WT cells (Figure S7D). This change is consistent with the reduction in translation efficiency or mRNA loss in WT cells.

Taken together, these results show a potential role of RNase L in inhibiting the translation of *GADD34*, thus modulating the ISR during the innate immune response, and suggest that RNase L may have a broader role in regulating translational control (Figure S7E).

## DISCUSSION

In this study, we examined how direct RNase L activation by 2-5A, and attendant global RNA degradation in the cell, affects gene expression. Our work shows that the global loss in mRNA is compensated for by increases in transcription, particularly via activation of ZAKα, and tuned by changes in translation.

We showed that one way mRNA cleavage leads to new transcription is via activation of the RSR. RNase L activation turns on the p38 and JNK MAPK pathways, independent of other dsRNA sensors that are activated by poly(I:C) (Figure 1). We established that ZAKα can be triggered directly by RNase L activity but not by the other dsRNA-sensing pathways induced by poly(I:C). Moreover, it is likely that other mechanisms, beyond the RSR, exist to activate transcription in response to global RNA degradation. For instance, global RNA loss in the cytoplasm was proposed to free up RNA-binding proteins that, in turn, modulate transcription due to their relocation to the nucleus.^48^ Importantly, we demonstrated that, despite widespread mRNA decay, the cell retains the capacity to increase production of proteins, particularly transcription factors (Figure 1D). While we previously found that the peptides derived from altORFs on mRNA fragments could not be readily detected,^25^ our observation of ZAKα activation suggests that fragment translation could be important for triggering ribosome stalling and potentially collisions.

Ribosome collisions are known to form when a ribosome stalls during elongation and upstream ribosomes bump into it.^49^ One mechanism to account for their occurrence is that mRNAs undergoing active translation are cleaved by RNase L, resulting in the ribosomes stalling whenever they reach a 3′ end of a 5′ cleavage fragment (Figure 1F). Prior work using ribosome profiling demonstrated this kind of 3′ end stalling and formation of ribosome collisions when the homologous nuclease, Ire1, cleaves mRNAs during the UPR in fission yeast.^30^ Consistent with the idea of an increase in ribosome collisions during viral infection, it was shown that vaccinia virus infection leads to an increase in ubiquitination of the ribosomal protein uS10 by the ubiquitin ligase ZNF598, which is known to be directly activated by ribosome collisions.^50^ Alternatively, ribosome collisions could form for other reasons, including the widespread translation of alternative (non-canonical) ORFs, which we previously reported,^25^ since such ORFs may be less evolutionarily tuned to minimize slow translation.^51^ We also note that ZAKα has been reported to be somewhat activated by individual stalled ribosomes, implying that ribosome collisions are not strictly required for the effects observed here.^52^ It is also possible that ZAKα could be activated in other ways that involve the cleaved RNA fragments through a yet-unknown mechanism. The effect of RNase-L-induced activation of the RSR was previously shown to be JNK-mediated programmed cell death in response to viral infections.^18^ Interestingly, both ZAKα and RNase L activation was shown to trigger the inflammasome independently.^24,53^ Considering our data, it is plausible that RNase L also triggers inflammasome activation through ZAKα and contributes to cell death.

Previous RNA-seq studies hinted that RNase L activation may act together with the other dsRNA-sensing pathways to amplify the innate immune response.^17^ Our findings here confirm this synergy by showing that activation of JNK and p38 occurs by both RNase-L-dependent and independent routes. Since impairment of RNase L activation can have serious health consequences linked to a reduced ability to fight viral infections,^54,55^ it is possible that the loss of this two-pronged activation weakens other branches of the immune response that are independently triggered by dsRNA. In addition, our observation that ERK is not activated and p38 is activated by 2-5A has not been consistently observed in previous studies,^14,19^ further suggesting that the activation of kinases by RNase activity is regulated by other factors, possibly in a cell-line-dependent way.

Interestingly, our data also suggest that RNase L may lead to further regulation of eIF2α kinase activity. Our analysis of eIF2α phosphorylation levels in cells treated with poly(I:C) shows that phosphorylation is higher when RNase L is active. This is consistent with previous reports showing that, in addition to PKR, another kinase can phosphorylate eIF2α during poly(I:C) treatment, and it is dependent on RNase L activity.^17^ A possible explanation is that RNase L activates the eIF2α kinase GCN2, which is sensitive to ribosome collisions^21,56–58^ and enhanced by ZAKα activation.^21^ Intriguingly, our analysis and previous studies^5,25^ suggest that direct RNase L activation via 2-5A does not activate any kinase of eIF2α. Therefore, enhanced phosphorylation of eIF2α when RNase L is active likely relies on additional factors that are activated by poly(I:C) but are absent when only RNase L is active.

Beyond control of new transcription, we also examined roles for RNase L in changing which transcripts are translated. Intriguingly, RNase L may somewhat limit the eIF2α-mediated translational activation of *GADD34*. This effect would strengthen the known inhibition of this gene due to its susceptibility to RNase-L-mediated degradation (Figure 6).^17^ One possible explanation for this apparent inhibitory effect of RNase L on the translation of this gene is that the availability of eIF2α may increase when RNase L is active due to the general reduction in demand for translation, as noted above. As a result, the impact of phosphorylation of eIF2α on translation initiation may be lessened, thus preventing the shift from uORF to main ORF translation on these transcripts. Alternatively, since RNase L cleaves rRNAs, it is plausible that this damage could alter the choice of start codon (uORF vs. main ORF) by scanning 40S subunits. Another possibility is that, as we previously showed,^25^ RNase L activation leads to an increase in the proportion of ribosome footprints in 5′ UTRs globally, and this holds true for GADD34 (Figures 6B and 6C). This effect may arise from direct initiation on mRNA cleavage fragments generated when RNase L cleaves within the 5′ UTR. This change in initiation could therefore lead to the reduced translational activation of *GADD34*. Interestingly, we also found that eIF2α phosphorylation levels were decreased in poly(I:C)-treated RNASEL-KO cells compared to WT. Since GADD34 is a phosphatase of eIF2α,^59,60^ we postulate that increased amounts of GADD34 protein in poly(I:C)-treated *RNASEL*-KO cells could contribute to the decrease of eIF2α phosphorylation.

In addition, we found that RNase-L-dependent changes in translation efficiency of *IFIT1–3* are correlated with an apparent shift in uORF to main ORF translation. One potential explanation for this effect is that these *IFIT* genes, much like *GADD34*, could be activated by the ISR. In this scenario, RNase L would similarly serve to limit their translational activation in addition to their likely downregulation at the mRNA level via degradation. Such dampening could be important for limiting autoimmune conditions. Hence, we postulate that overactivation of RNase L in autoimmune disease could contribute to an enhanced activation of the ISR. For example, impairments in the OAS-RNase L pathway are one of the contributing factors in MIS-C (multisystem inflammatory syndrome in children).^61^ Furthermore, both RNase L deficiencies and overactivation of RNase L are linked to inflammatory bowel disease.^7,10,62^ Thus, active RNase L may serve a dual role in innate immunity, activating some pathways while counter-balancing others.

Finally, we found that the generic endonuclease RNase A evokes a transcriptional response, including that mediated by ZAKα, that is very similar to that of RNase L (Figure 3). In addition, RNase A, or the ISR-mediated endonuclease IRE1, can also trigger the translation of altORFs, much like RNase L (Figure 4). This evidence suggests that other endonucleases, such as the viral endonuclease Nsp1 or the apoptotic host endonuclease EndoU,^63–65^ could potentially cause outcomes similar to that of RNase L. Since both viruses and their hosts express endonucleases, the ultimate effects of generic endonuclease activation may be tunable to benefit either the virus or the host.

Overall, our data offer a model in which activation of RNase L induces ribosome stalling and/or collisions, leading eventually to cell death via key transcriptional pathways (Figures 1F and 2D) related to the kinase ZAKα. This suggests that ZAKα could be a potential target for therapeutics aimed at modulating the effects of RNase L. This is especially relevant since RNases are already being used or tested for cancer therapy, such as RNase A.^66,67^ We speculate that a component of the mechanism behind these RNase therapies could result from ZAKα activation via ribosome stalling and/or collisions that occur as a result of RNase activity in the cytoplasm. Thus, modulating the levels of ZAKα activation could be the key to the safety and efficacy of these treatments. In addition, RNase L may have other roles in affecting the ISR via translation of ISR target genes. The effects of RNase L on transcription and translation may be important for reinforcing or dampening effects evoked by other dsRNA sensors, pointing to linkages between pathways in the innate immune response. Further study is needed to dissect these connections that bring together the entire spectrum of mRNA activities, including synthesis, translation, and degradation.

### Limitations of the study

We found that activation of RNase L or a generic endonuclease, such as RNase A, triggers the kinase activity of ZAKα, a known sensor of stalled or collided ribosomes. However, the exact mechanism for how mRNA fragmentation causes ribosomes to stall, and whether this includes formation of collisions, remains ambiguous. In addition, we activated RNase L via transfection of 2-5A or poly(I:C) and observed downstream signaling via ZAKα. It remains to be established whether activation of RNase L due to viral infection or induction of cancer evokes the same outcomes.

## STAR★METHODS

Detailed methods are provided in the online version of this paper and include the following:

### RESOURCE AVAILABILITY

#### Lead contact

Further information and requests for resources and reagents should be directed to and will be fulfilled by the lead contact, Nicholas Guydosh (nicholas.guydosh@nih.gov).

#### Materials availability

Plasmids and other non-commercial reagents are available upon request.

#### Data and code availability

- All raw sequencing (fastq) and processed alignment (wig) files are available at NCBI GEO in record GEO: GSE244176 (ribosome profiling) and GEO: GSE244125 (RNA-seq).
- Custom code used for ribosome profiling in this study is available on Github: https://github.com/guydoshlab. Gene expression analysis workflow is available on Github: https://github.com/TriLab-bioinf/TK_65_RNAseL_manuscript. DOI link available here Zenodo: https://doi.org/10.5281/zenodo.11093742.
- Any additional information required to reanalyze the data reported in this work paper is available from the lead contact upon request.

### EXPERIMENTAL MODEL AND STUDY PARTICIPANT DETAILS

WT and *RNASE* L KO human A549 lung carcinoma cells (male) were cultured in RPMI (Gibco Cat # 60870127) complemented with 10% Fetal Bovine Serum (Gibco FBS). Human MCF10A cells (WT and *ZAK* KO, female) were cultured in DMEM/F12 Ham’s Mixture supplemented with 5% horse serum, 20 ng/mL EGF, 0.5 mg/mL hydrocortisone, 100 ng/mL cholera toxin, and 10 μg/mL insulin and 2mM glutamine. Cells were incubated at 37°C in the presence of 5% CO_2_. KO cell lines were confirmed by western blotting for the protein encoded by the target gene. A549 and MCF10A cell lines used in this study were a kind gift of Dr. Bernie Moss (NIH) and Dr. Colin Wu (NIH), respectively, and generated as described in.^21,68^

### METHODS DETAILS

#### Cell culture

Cells used were tested and negative for Mycoplasma contamination throughout the study. Mycoplasma testing was performed using eMyco Valid Mycoplasma PCR detection kit (BioLink) or ATCC Universal Mycoplasma Detection Kit. Images of cells were obtained on an EVOS FL microscope.

#### rRNA cleavage assay

Total RNA was extracted from ~3×10^5^ cells using Direct-zol mini kit (Zymo) or Trizol reagent (Invitrogen) according to the manufacturer’s protocol. The amount of total RNA was computed by absorbance at 260 nm measured by NanoDrop (Thermo Fischer Scientific) and then diluted to 50–500 ng/μl. Then RNA samples were run on a Bioanalyzer 2100 (Agilent) using the RNA 6000 Nano kit (Agilent) or TapeStation with the Agilent RNA ScreenTape assay. Minute run-to-run shifts in band sizes are due to inherent limitations of the instrument.

#### Sample preparation for RNA-seq and ribosome profiling

Cells used in experiments were grown until they reached near confluency (70–90% confluent) in a T75 flask. Then cells were transfected with the pre-incubated mixture of 1 μM 2-5A or 0.25 μg/ml poly(I:C) (Invitrogen, low molecular weight) and Lipofectamine 3000 (7.5 μl/6-well) in Opti-Mem serum free media as recommended by the manufacturer for 4.5 hours. Control cells (definied as untreated or −2-5A) were transfected with lipofectamine only. 2-5A was synthesized as described here.^25^ For a previous study, 2-5A was synthesized enzymatically *in vitro* by recombinant human OAS1 (reference) and used again here. Briefly, recombinant His-tagged OAS1 (p42) was expressed in BL21(DE3) *E.coli* as described before^69^ or with an alternative protocol using autoinduction media.^70^ Then cells were collected by centrifugation at 7000 g for 15 min at 4°C. Cells were lysed in B-per protein extraction reagent 4 ml/gram bacterial pellet in the presence of cOMPLETE mini protease inhibitor cocktail (Roche) for 15 min at room temperature. Next, bacterial lysate was cleared by centrifugation at 34,000 g for 1 hour at 4°C. The supernatant was filtered (45 μm pore size filter, Millipore) and loaded onto a HisTrap (GE Healthcare) nickel column. The column was next washed with wash buffer (20 mM Hepes (pH 7.5), 300 mM NaCl, 10% (vol/vol) glycerol, 1 mM TCEP and 50 mM imidazole) and OAS1 was gradient eluted with 500 mM imidazole. Fractions were evaluated by SDS-PAGE. Then OAS1 containing fractions were pooled, concentrated and exchanged (Zeba Spin desalting Column, 7K MWCO, Thermo Scientific) into storage buffer (20 mM Hepes (pH 7.5), 300 mM NaCl, 10% (vol/vol) glycerol and 1 mM TCEP). Final protein preparation was stored at −80°C. Concentration of OAS1 was determined by using NanoDrop Spectrophotometer (Thermo Fischer Scientific) (Mw = 41.5 g/mol and *ε* = 65,485 M^−1^cm^−1^). To produce 2-5A, 2 μM purified OAS1 was incubated with 1.25 OD_260_ poly(I:C) and 10 mM ATP, 20 mM Hepes (pH 7.5), 50 mM NaCl, 30 mM MgCl_2_, 10% (vol/vol) glycerol, 4 mM DTT at 30°C for 2 hours. To stop the reaction, samples were incubated at 85°C for 15 minutes. Then the samples were filtered (22 μm pore size Millipore filter) and the different 2-5A species were separated on a 16/10 MonoQ column as described before.^69^ The same 2-5A fractions from several runs were pooled and run on 16/10 MonoQ column to achieve higher concentrations. 2-5A concertation was estimated by NanoDrop Spectrophotometer at 259 nm. Then yielded 2-5A was aliquoted and stored at −80°C.

For electroporation of RNase A or BSA, confluent T75s (~6–8×10^6^ cells) were trypsinized and resuspended in MaxCyte electroporation buffer and electroporated in 3XOC25 cuvettes (MaxCyte) in the presence of 160 ng/ml RNase A or BSA with standard settings for A549 in the MaxCyte ATX electroporator. Cells were plated on T75 flasks and collected after 4.5 hours incubation. Then cell lysates were prepared and collected as for ribosome profiling.^25^ The same samples were split for RNA-seq and ribosome profiling.

#### Ribosome profiling

Ribosome profiling of RNase A and BSA electroporated cells was carried out as previously reported,^25^ except that rRNA depletion was carried out by siTools riboPool rRNA depletion kit designed for human ribosome profiling. The ribosome profiling data was processed and signatures of altORF translation were assessed as before.^25^ Sequencing was performed on HiSeq 3000 (single end 50) at the NHLBI or NIDDK DNA Sequencing and Genomics Core. These ribosome profiling data and previously published data were used for analysis of translation efficiency (see below).

#### Total RNA-seq

Total RNA was extracted from 100 μl of lysates prepared for ribosome profiling using Direct-zol mini kit (Zymo) or Trizol reagent (Invitrogen) according to the manufacturer’s protocol. The amount of total RNA was computed by absorbance at 260 nm measured by NanoDrop (Thermo Fischer Scientific) and then diluted to 50–500 ng/μl. Then RNA samples were run on Bioanalyzer 2100 (Agilent) using the RNA 6000 Nano kit (Agilent). Samples for sequencing were subject to rRNA depletion and library preparation by Illumina Stranded Total RNA Prep kit (#20040529) or Truseq Stranded Total RNA Prep (#20020596). Sequencing was performed on HiSeq 3000 or Novaseq 6000 (paired end 50 or 75 cycle) at the NHLBI DNA Sequencing and Genomics Core.

#### Computational analysis

##### Processing and alignment of RNA-seq reads

Data files from libraries run in multiple lanes were combined. After removing residual adapters from sequencing reads with the bbduk.sh tool from bbtools (v37.62), sequencing data were mapped to a database of highly abundant rRNA and tRNA sequences with bowtie2 (v2.4.5).^71^ Unmapped reads were then mapped to the human reference genome (GRCh38) with STAR (v2.7.10a) using default parameters except for alignSJDBoverhangMin = 1, outFilterMismatchNmax = 2, alignEndsType = EndToEnd, and leaving multimaper reads mapping to less than 11 loci.^72^ Duplicated reads were removed with picard MarkDuplicates (v2.26.10)^73^ and quantification of fragments per gene was carried out with the featureCounts tool from Subread software (v2.0.1)^74^ using Ensembl gene annotations (Ensembl version hg38) by counting read-pairs falling within coding regions (CDS) as fragments and adding fractional counts for multimapper reads. BigWig files were generated with bamCoverage tool from DeepTools (v3.5.1).^75^

##### Analysis of RNA-seq data

Processed bulk RNaseq data was analyzed in R using the following workflow on Github: https://github.com/TriLab-bioinf/TK_65_RNAseL_manuscript. Differential gene expression analysis was performed with the R package DESeq2 (v1.34.0).^27^ We note that we identified “batch effects” (non-biological experimental factors) based on the initial analysis of PCA plots and hence we used an interaction term to improve our analysis with DEseq2 (see workflow above). Volcano plots show DE genes as *p*_adjusted_ value <0.05, log_2_fold change >1. GO enrichment analysis were carried out with the gseGO function from the R package ClusterProfiler (v4.2.2).^76^ 5 out of 15 categories with the highest Normalized Enrichment Scores are shown in Figure 1 where *p*_adjusted_ <0.05. Categories not shown had highly overlapping gene sets with those that are highlighted.

To identify genes associated with JNK/p38 and interferon in Figures 2C and 3C, we utilized gene collections from Harmonizome 3.0^77^ that were based on Gene References Into Function (GeneRIFs, JNK, p38 datasets, Table S3). Within these datasets, we used a subgroup of genes that were upregulated with 2-5A treatment (JNK/p38 plots, *N* = 54, Table S3) to create violin plots in Figures 2D and 3D. For interferon-related genes in Figure 2, we used the hallmark datataset from the Molecular Signature Database^78^ (“interferon-gamma-response,” Table S3) for a subgroup of genes that were upregulated in poly(I:C) treated WT cells (interferon-related plots, *N* = 61, Table S3). We performed paired t-test to determine significant differences between the groups within each plot. Statistical testing (paired t-tests) showed significant differences between data sets as indicated (*p* value <0.001, marked by ***) in Figure 2D. To perform correlation analysis between datasets from cells treated with 2-5A and poly(I:C), we performed a sequential filtering process to compare different subsets of genes according to differential expression status determined by p value only (see R workflow).

##### Processing of ribosome profiling footprint data

Ribosome profiling data was processed as before to remove linkers and duplicates^25^ and is briefly described again here. The fastq files were de-barcoded by the core facility and footprints of 25–34 nt were sorted by internal sample barcode and trimmed of linkers by Cutadapt. Any contaminating tRNAs and rRNAs were filtered out by Bowtie1 allowing two mismatches in -v mode. We created the noncoding RNA fasta file by downloading rRNA sequences from the SILVA project (release 128)^79^ and tRNA sequences from GtRNAdb (*H. sapiens* release 16).^80^ Next, an in-house python script (dedup) was used to remove PCR duplicates by comparing the 7 nucleotide unique molecular identifiers (UMIs) in libraries where they were incorporated. Then UMIs were trimmed by Cutadapt. After processing, reads were 5’ aligned with STAR (v2.8.2a) to the human genome (hg38, UCSC). Multimapping was allowed up to 200 times (–outFilterMultimapNmax option) and the maximum mismatch/read was set to 1 (–outFilterMismatchNmax option). For both the RNA-seq and ribosome profiling data CDS read counts were generated by featureCounts tool from Subread software (v2.0.1) including multimappers as fractional counts and reads on exon-exon junctions (-M –fraction and -J options, respectively). Note that files (fastq and wig) for four ribosome profiling datsets consist of data from technical replicates were combined and analyzed together after initial processing to remove linkers and duplicates. These are indicated with “F2” in the name of the file.

##### Analysis of translational efficiencies

We used our DESeq2 pipeline as described above for RNA-seq to obtain differential expression values (log2 fold changes) for the ribosome profiling experiments. These changes (RNA-seq and ribosome profiling log2 fold change values) were plotted against each other in Figure 5A to visualize where translation efficiency (TE) was changing. We used a filter for log2 fold changes >2 and *p* adjusted value <0.01 as the definition for points we considered for TE changes for all datasets. We further filtered these sets for normalized mean counts across the samples >50 to eliminate low expressing genes. Of this reduced dataset, we excepted TE changes >2 (defined by dotted lines on Figure 5A) for further consideration. The output of the DESeq2 analysis for the RNA-seq and ribosome profiling are provided as Tables S1 and S2. For further analysis of individual genes, we computed the TE directly by dividing reads per million (rpm) values for ribosome profiling and RNA-seq data that mapped to the coding sequence. This allowed us to directly compare replicates within a treatment group.

##### Analysis of translation for altORF analysis and individual genes

For more detailed analysis of ribosome profiling data, we implemented a reduced-transcriptome alignment and python analysis pipeline also described here.^25,81^ We used the Ref-Seq Select+MANE (ncbiRefSeqSelect), as downloaded from UCSC on 14 April 2020, for this alignment after removal of duplicates on alt chromosomes. In detail, we took reads that had been digitally subtracted for non-coding RNA and aligned them to a transcriptome with bowtie version 1.2.3 using the parameters -v 1 (1 mismatch allowed) and -y. Then, using our python tools (writegene2) to output reads for individual gene tracks, we then computed the ratio of rpm values that mapped to the UTR to those that mapped to the main ORF (Figures 6, S6, and S7), based on the defined annotations. When uORFs overlapped the main ORF for *ATF4*, the overlap region was assigned to the UTR for the calculation of UTR to ORF ratio. We also assessed global UTR:ORF ratios with boxplots (genelist_m function) or metagene plots (metagene_m function) (Figures 4 and S5). In general, we assumed a shift of 12 nt between the 5’ end of a read and the P site of the ribosome. Data are displayed in individual tracks to correspond with ribosome P sites. For metagene and individual gene tracks, representative replicates are shown.

#### Western blotting

Protein levels were assessed by SDS-PAGE electrophoresis and immunoblotting during 2-5A and poly(I:C) treatment. For western blotting cells were grown on a 6-well plate up to ~70% confluency then transfected with 2-5A or poly(I:C) as described above. Cells were trypsinized after 4.5 hours (Gibco TrypleExpress) and collected by centrifugation at 200 g for 5 minutes. The cell pellet then was resuspended in 90 μl of 10 mM Tris-HCL pH = 8.0, 150 mM NaCl and 1 % triton-X and incubated on ice for 5 minutes. Samples were then centrifuged for 1500 rpm, 4 minutes at 4°C. The supernatant was then mixed with equal amount of 2X SDS-PAGE buffer (Quality Biological) and samples were sonicated with a Brenson sonicator for 10–15 seconds until the sample was no longer viscous. 5–10 μl of sample was run on a 4–20% gradient Mini-PROTEAN Tris-HCl gel (BioRad) or 7% PhosTag (Wako) gel prepared according to manufacturer’s instruction and as described here.^21^ Proteins were transferred to a 0.2 μm PVDF membrane using the Trans-Blot Turbo System (BioRad, 1.3 A, 7 minutes for 4–20% gradient Mini-PROTEAN Tris-HCl gel or 1.3 A for 14 minutes for PhosTag gel). Membranes were blocked in EveryBlot Blocking Buffer (BioRad) for 10 minutes at room temperature. This was followed by incubation with primary antibodies overnight in TBST (Tris-buffered saline plus 0.1% tween) and 1% milk. The following antibodies were used: anti-p38 MAPK (Cell signaling Technologies #9212, 1:2000 dilution), anti-P-p38 MAPK (Cell signaling Technologies, 1:2000 dilution, #9211), anti-IRF3 (Cell signaling Technologies #11904), anti-P-IRF3 Ser396 (Cell signaling Technologies #4947), anti-ZAK (Bethyl A301-993A), anti-P-JNK (Thr183/Tyr185) (Cell signaling Technologies #4668), anti-JNK (Cell signaling Technologies #9252), anti-p65 (Cell signaling Technologies #8242), anti-ERK1/2 (Cell signaling Technologies #4695), anti P-ERK1/2 (Cell signaling Technologies #4370), anti-IFIT1 (abcam #305301), anti-IFIT2 (abcam #305231), anti-GADD34 (Cell signaling Technologies, #41222), anti-eIF2α (Bethyl, #A300-721A), anti-P-eIF2α (abcam #32157), anti-cofilin (abcam #124979), ant-caspase-3 (Cell signaling Technologies, #9662). After washing 3 times for 5 minutes in TBST, secondary antibodies were incubated with the PVDF membrane for 1 hour at room temperature (Goat anti-rabbit antibody, BioRad, 1:3000). Then, after additional washes (3 times, 5 minutes each) in TBST, the PVDF membrane were incubated with Clarity Western ECL substrate (BioRad) for 5 minutes and proteins were visualized by Amersham Imager 600. Western blots were repeated for 2–7 times using biological replicates unless indicated otherwise and blots were quantified using Fiji.^82^ Replicate images are be provided on Mendeley Data (see additional resources).

### QUANTIFICATION AND STATISTICAL ANALYSIS

#### Counts of biological replicates are denoted by n in the figure legends

##### Ribosome profiling and RNA-seq

Experiments were repeated on distinct samples (biological replicate) at least twice (Figures 3, 4, S4, and S5) (*n* = 2) or 3–5 times (Figures 1, 2, 5, 6, S1, S6, and S7). Each biological replicate dataset was analyzed individually for consistency. Analysis and comparison of all datasets are provided in the main and in the supplemental figures. Correlation analysis (Pearson’s r of log-transformed data) was performed by R Studio (see R code). Statistical analysis of violin plots (Figure 2D) is described under “Analysis of RNA-seq data”. Calculation of translational efficiencies are described under “Analysis of translational efficiencies”.

##### Western blotting

Western blotting experiments were carried out 3–7 times, except in Figures S3, S2C, S2E, and S2G (single experiment) and S7D (two replicates). Quantification in Figure 2F was carried out by Fiji as described here: https://www.navbo.info/DensitometricAnalysys-NIHimage.pdf.

Biorender was used to create some figure panels.

### ADDITIONAL RESOURCES

Western images and replicates, Mendeley Data: https://doi.org/10.17632/dkxwy455z3.1

## Supplementary Material

1

2

3

4

## Figures and Tables

**KEY RESOURCES TABLE:** 

REAGENT or RESOURCE	SOURCE	IDENTIFIER

Antibodies		

p38 MAPK antibody	Cell signaling Technologies	Cat# 9212; RRID:AB_330713
P-p38 MAPK antibody	Cell signaling Technologies	Cat# 9211; RRID:AB_331641
IRF3 antibody	Cell signaling Technologies	Cat# 11904; RRID:AB_2722521
P-IRF3 Ser396 antibody	Cell signaling Technologies	Cat # 4947; RRID:AB_823547
ZAK antibody	Bethyl	Cat# A301-993A; RRID:AB_1576612
JNK antibody	Cell signaling Technologies	Cat# 9252; RRID:AB_2250373
P-JNK (Thr183/Tyr185) antibody	Cell signaling Technologies	Cat# 4668; RRID:AB_823588
p65 antibody	Cell signaling Technologies	Cat# 8242; RRID:AB_10859369
ERK1/2 antibody	Cell signaling Technologies	Cat# 4695; RRID:AB_390779
ERK1/2 antibody	Cell signaling Technologies	Cat# 4370; RRID:AB_2315112
IFIT1 antibody	Abcam	Cat# 236256; RRID:AB_3094695
IFIT2 antibody	Abcam	Cat# 305231; RRID:AB_3094696
GADD34 antibody	Cell signaling Technologies	Cat# 41222;
eIF2α antibody	Bethyl	Cat#A300-721A
P-eIF2α antibody	Abcam	Cat# ab32157; RRID:AB_732117
Cofilin antibody	Abcam	Cat# ab124979; RRID:AB_10972347
Histon H3 antibody	Abcam	Cat# ab1791; RRID:AB_302613
Caspase-3 antibody	Cell signaling Technologies	Cat# 9662; RRID:AB_331439

Chemicals, peptides, and recombinant proteins		

Cycloheximide	Sigma	C7698
B-Per protein extraction reagent	Thermo Fischer Scientific	78248
RNase I	Ambion	AM2294
Turbo DNase (2U/μl)	Thermo Fischer Scientific	AM2239
PNK	NEB	M0201L
T4 RNA ligase 2, truncated K227Q	NEB	M0351L
5’ deadenylase	NEB	M0331S
RecJ exonuclease	Biosearch Technologies	RJ411250
Superscript III Reverse Transcriptase	Invitrogen	18080044
Phusion DNA polymerase	ThermoFisher Scientific	F530L
B-Per reagent	ThermoFisher Scientific	78248
Isopropyl β-D-1-thiogalactopyranoside	Sigma	I5502
cOMPLETE mini protease inhibitor cocktail	Sigma	11836153001
Poly I:C Low Molecular Weight	Invivogen	tlrl-picw
Lipofectamine 3000	ThermoFisher Scientific	L3000008
EGF	PeproTech	AF-100-15
Hydrocortisone	Sigma	H4001-5G
Insulin	Sigma	I1882-100MG
Cholera toxin	Sigma	C8052-.5MG
PhosTag Acrylamide 5 mM aqueous solution	Wako-Fijifilm	AAL-107S1

Critical commercial assays		

Stranded Total RNA Prep kit	Illumina	20040529
TruSeq Stranded Total RNA Library Prep Kit	Illumina	20020596
Mycoplasma PCR detection kit	BioLink	13-1100
ATCC Universal Mycoplasma Detection Kit	ATCC	30-1012K
RNeasy RNA miniprep kit	Qiagen	74104
Direct-zol RNA miniprep	Zymo	R2051
siTools rRNA removal kit human	siTools	dp-K012-000042
CircLigase ssDNA ligase kit	Biosearch Technologies	CL4115K
5’ DNA adenylation kit	NEB	E2610L
Oligo clean & concentrator kit	Zymo Research	D4060
Agilent RNA ScreenTape components	Agilent	5067-5576 5067-5577 5067-5578
Agilent D1000 DNA high sensitivity	Agilent	5067-5583
SreenTape components		5067-5582
Agilent RNA 6000 Nano Kit	Agilent	5067-1513
Agilent high sensitivity DNA kit	Agilent	5067-4626

Deposited data		

Raw and analyzed data	This paper and Karasik et al.^25^	GEO: GSE244176, GSE244125
Raw western blot gel images	This paper, Mendeley data	Mendeley Data: https://doi.org/10.17632/dkxwy455z3.1

Experimental models: Cell lines		

MCF10AWT	Wu et al.^21^	N/A
MCF10A *ZAK* KO	Wu et al.^21^	N/A
A549 lung carcinoma cell line; wild type	Liu and Moss^68^	N/A
A549 lung carcinoma cell line; *RNASEL* KO	Liu and Moss^68^	N/A

Oligonucleotides		

Recombinant DNA		

pcDNA5-RNASEL-WT	Karasik et al.^25^	N/A
pcDNA5-RNASEL-H672N	Karasik et al.^25^	N/A
OAS1 plasmid	Poulsen et al.^69^	N/A

Software and algorithms		

Bowtie	SourceForge	1.3.1
STAR	bioconda	2.7.8a and 2.7.10a
Prism	GraphPad	8.1.0
RStudio	Rstudio, Inc	2023.06.0+421
samtools	bioconda	1.14
Custom python2 tools (genelist_m, metagene_m, writegene2_m)	GitHub	GuydoshLab
Custom python2 tool (dedup)	GitHub	GuydoshLab
cutadapt	GitHub	3.4 and 4.4
Subread	bioconda	2.0.1
BioRender	https://www.biorender.com	BioRender

Other		

DMEM/F-12	Sigma	15-090-CV
RPMI 1640 medium GlutaMax supplement	Thermo Fischer Scientific	61870036
Opti-MEM Reduced Serum Medium Glutamax supplement	Thermo Fischer Scientific	51985034
Dulbecco’s Phosphate-Buffered Saline	Thermo Fischer Scientific	14190250
Tryplate express	Thermo Fischer Scientific	12604-013
Fetal Bovine Serum, One Shot format	Thermo Fischer Scientific	A3160602
Horse serum	Thermo Fischer Scientific	16050-122

## Highlights

- Activated RNase L acts with dsRNA-sensing pathways to promote cell signaling
- RNA fragmentation induces transcription through ZAKα signaling
- Activation of RNase L modulates levels of eIF2α phosphorylation
- Translation of stress-responsive mRNAs is inhibited by active RNase L
