# Supplementary material for: Endonucleolytic RNA cleavage drives changes in gene expression during the innate immune response
Source: Cell Rep. Author manuscript; Available in PMC 2024 Jul 16. (PMC11251458; doi:10.1016/j.celrep.2024.114287)
Supplement: 1 [file NIHMS2005222-supplement-1.pdf]

Cell Reports, Volume *43*

Supplemental information

Endonucleolytic RNA cleavage drives changes in gene expression during the innate immune response

Agnes Karasik, Hernan A. Lorenzi, Andrew V. DePass, and Nicholas R. Guydosh

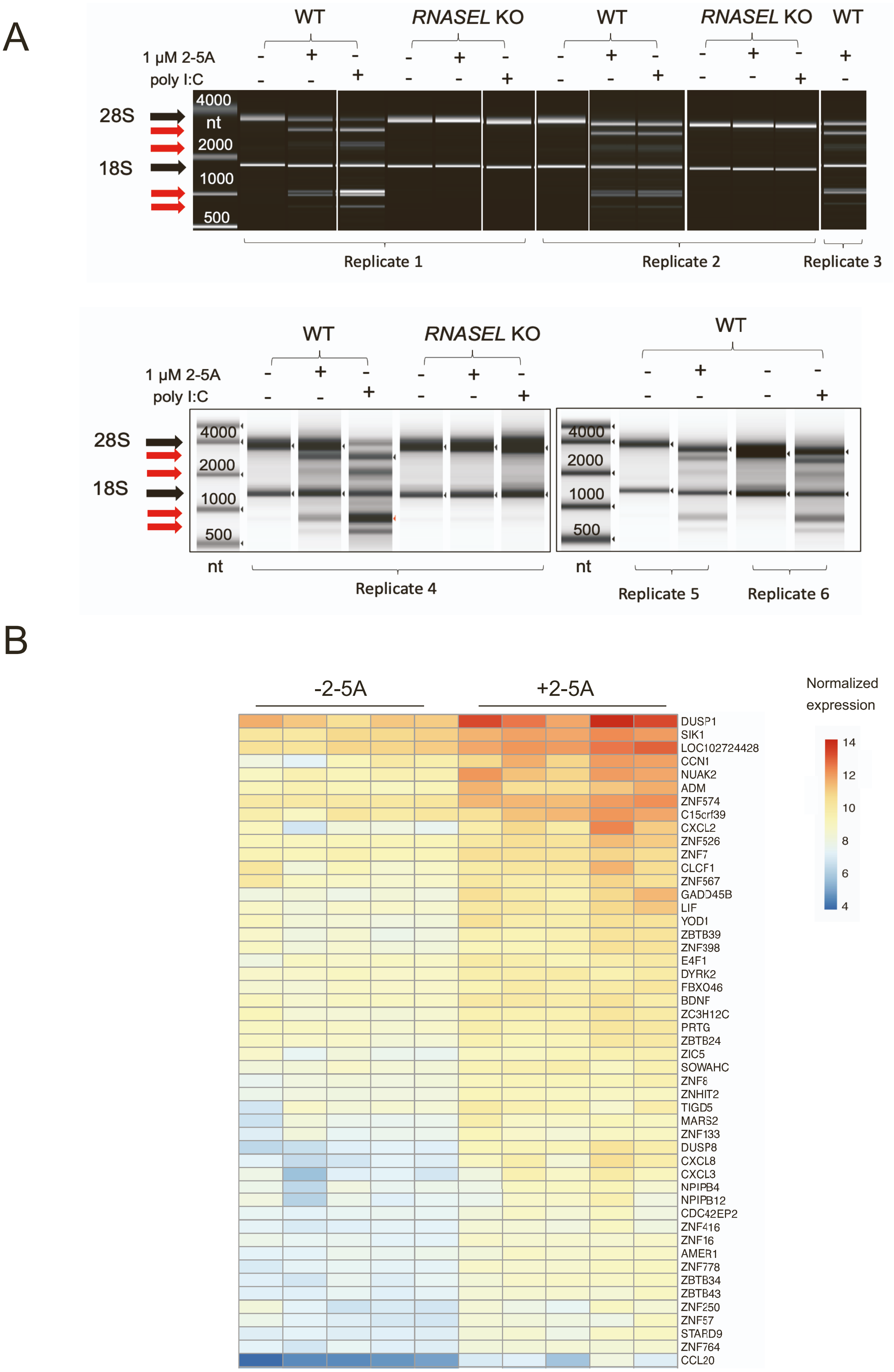
A
WT
RNASEL KO
WT
RNASEL KO
WT
1 µM 2-5A
poly I:C
28S
18S
4000
nt
2000
1000
500
Replicate 1
Replicate 2
Replicate 3
WT
RNASEL KO
WT
1 µM 2-5A
poly I:C
28S
18S
4000
2000
1000
500
nt
Replicate 4
Replicate 5
Replicate 6
B
-2-5A
+2-5A
Normalized expression
14
12
10
8
6
4
DUSP1
SIK1
LOC102724428
CCN1
NUAK2
ADM
ZNF574
C15orf39
CXCL2
ZNF526
ZNF7
CLCF1
ZNF567
GADD45B
LIF
YOD1
ZBTB39
ZNF398
E4F1
DYRK2
FBXO46
BDNF
ZC3H12C
PRTG
ZBTB24
ZIC5
SOWAHC
ZNF6
ZNHIT2
TIGD5
MARS2
ZNF133
DUSP8
CXCL8
CXCL3
NPIPB4
NPIPB12
CDC42EP2
ZNF416
ZNF16
AMER1
ZNF778
ZBTB34
ZBTB43
ZNF250
ZNF57
STARD9
ZNF764
CCL20

S1 Related to Figure 1. *RNase L activation leads to RNA fragmentation and gene expression changes.* **A** Cleavage patterns of total RNA extracts show activation of RNase L in 2-5A or poly(I:C) treated WT, but not *RNASEL* KO, A549 cells. rRNA cleavage assay performed on a BioAnalyzer (top) and TapeStation (bottom). Black arrows indicate the 28S and 18S rRNAs and red arrows show RNase L degradation products (n = 3-5). **B** Heatmap representation of the most highly expressed genes during RNase L activation. Trends are consistent across replicates.

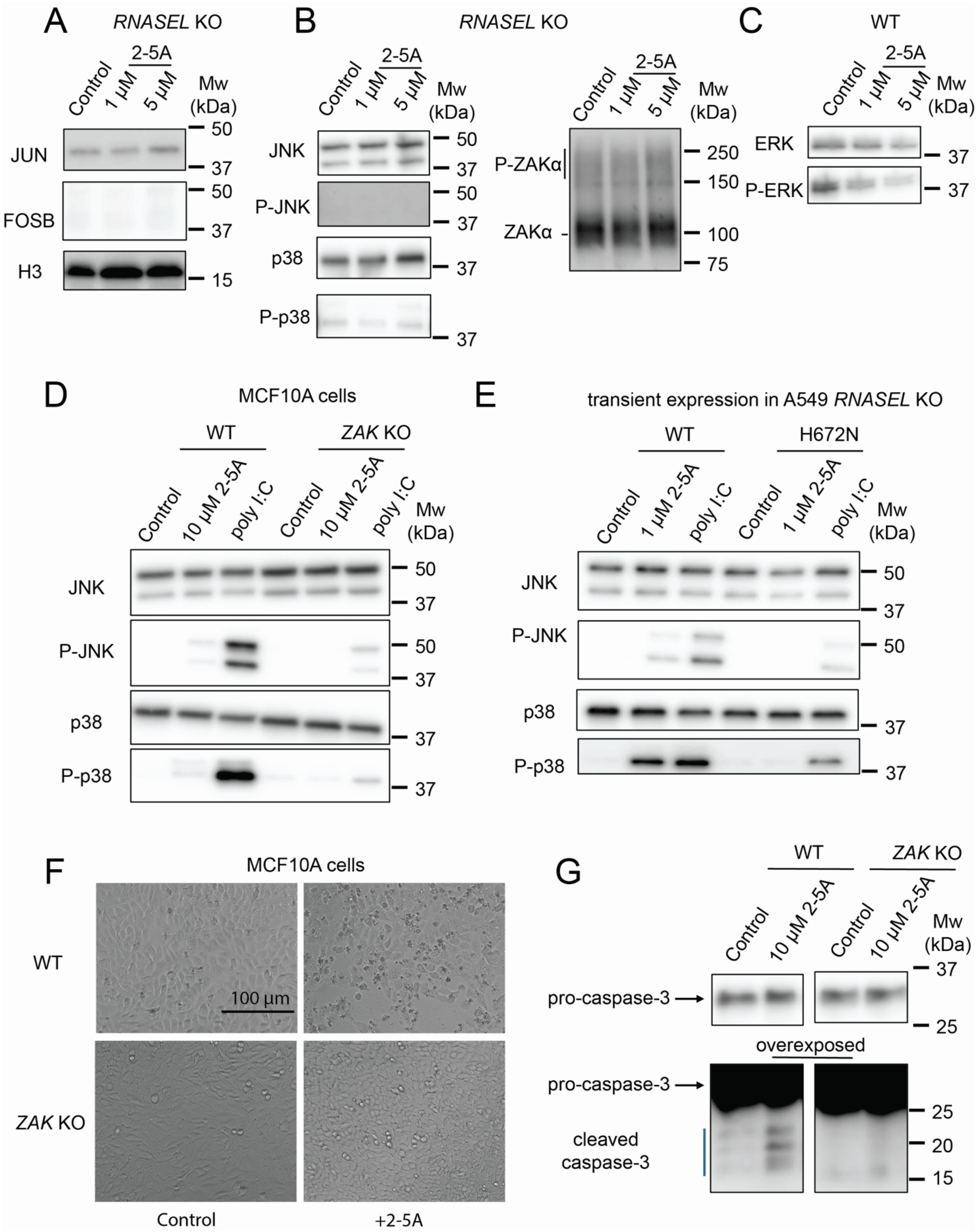
A
RNASEL KO
2-5A
Control
1 µM
5 µM
Mw (kDa)
JUN
FOSB
H3
B
RNASEL KO
JNK
P-JNK
p38
P-p38
P-ZAKα
ZAKα
C
WT
ERK
P-ERK
D
MCF10A cells
WT
ZAK KO
10 µM 2-5A
poly I:C
E
transient expression in A549 RNASEL KO
H672N
1 µM 2-5A
F
MCF10A cells
100 µm
Control
+2-5A
G
pro-caspase-3
overexposed
cleaved caspase-3

S2 Related to Figure 1. *RNase L activation leads to transcriptional changes through ZAKα, JNK and p38.* **A** Western blots show that transcription factors FOSB and JUN do not increase in *RNASEL* KO cell due to 2-5A activation (*n* = 4). **B** Western blots show JNK, p38 and ZAKα are not activated in *RNASEL* KO cells after 2-5A treatment (n = 3-7). **C** Western blots show ERK is not activated in WT cells after 2-5A treatment (*n* = 1). **D** p38 and JNK is phosphorylated and activated in MCF10A cells but not in *ZAK* KO (*n* = 3). **E** Catalytic activity of RNase L is required for activation of p38 and JNK (*n* = 1). H672N = catalytic mutant. **F** 2-5A treated cells show signatures of cell death in WT but not in *ZAK* KO cells after 16 hours of treatment (*n* = 2). Scale bar length is 100 µm. **G** Cleavage of pro-caspase-3 is increased in 2-5A treated WT but not in *ZAK* KO cells after 16 hours of treatment (*n* = 1).

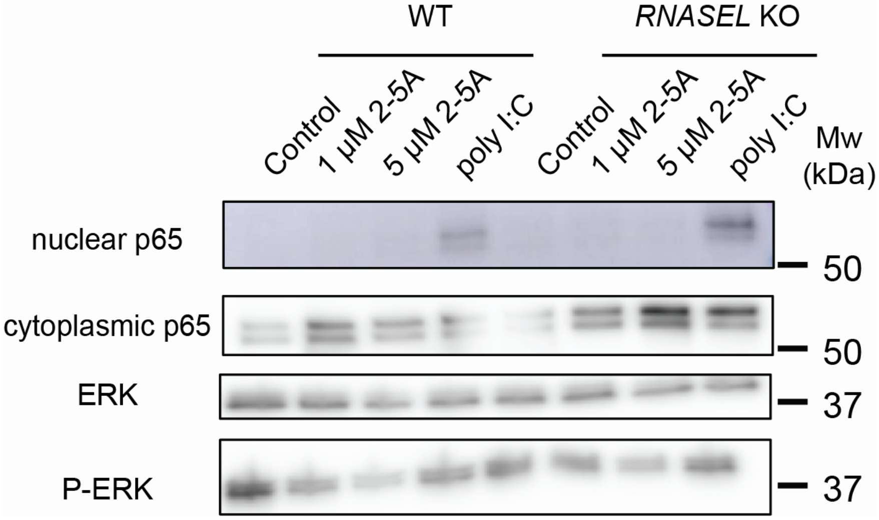

S3 Related to Figure 2. *poly(I:C) induces a proinflammatory response.* Western blots show no detectable effect of RNase L activation on NF-κB activation by poly(I:C) and no detectable activation by 2-5A. ERK activation was not observed during 2-5A (same blot section taken from S2C) or poly(I:C) treatment (*n* = 1).

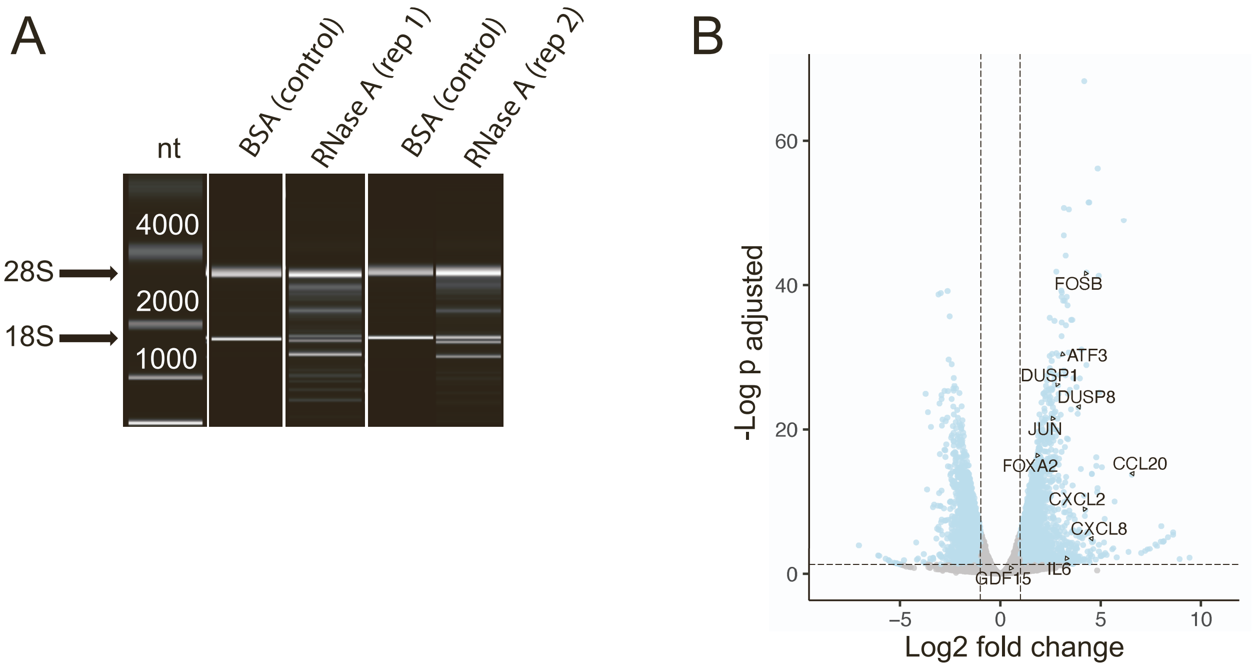

S4 Related to Figure 3. *Global RNA cleavage in the cell leads to transcriptional changes.* **A** Cleavage of RNAs in the cell occurs when RNase A is electroporated into cells, but not when BSA is electroporated, as observed by rRNA cleavage assays performed on BioAnalyzer. Arrows indicate the 28S and 18S rRNAs ($n = 2$). **B** Volcano plot of RNA-Seq data showing upregulation of example proinflammatory cytokines and transcription factors during RNase A electroporation. Differentially expressed genes define as $p_{adjusted}$ value <0.05, log_2fold change >1. Adjusted p values were calculated by the Benjamini-Hochberg method using DEseq2.

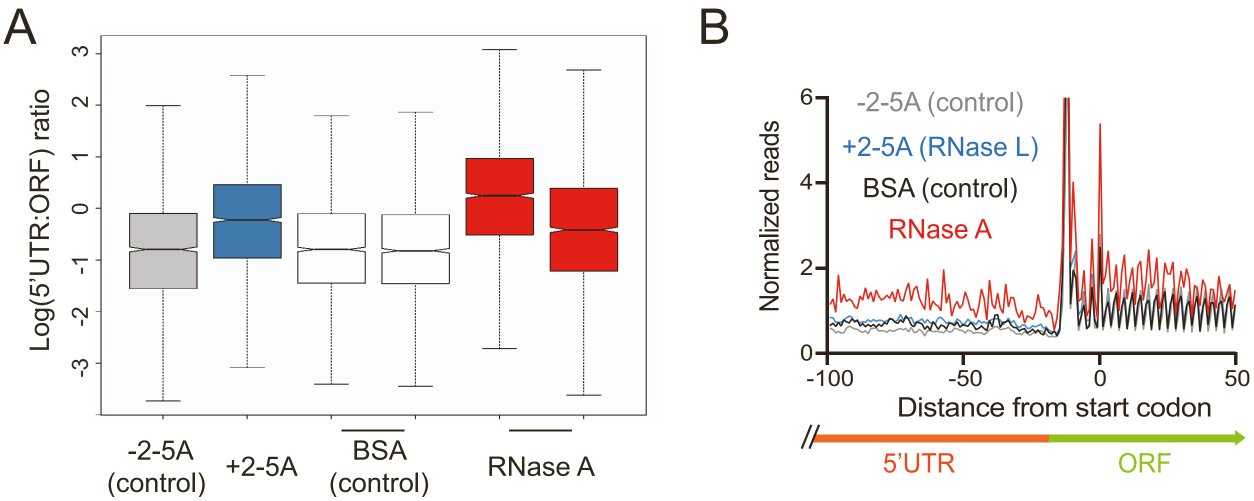

S5 Related to Figure 4. *RNA fragmentation leads to altORF translation*. **A** Increased 5'UTR:ORF ratios indicating higher relative uORF translation when active RNase is present in the cell, but not in controls (-2-5A and BSA electroporated). In box plots of UTR:ORF density ratios, boxes represent the interquartile range (IQR) and horizontal line is the median. Whiskers show 1.5*IQR and notches give $1.58*IQR/\sqrt{N}$. **B** Normalized average ribosome footprint occupancy (metagene plot) around the start codon of main ORFs reveals increased relative ribosome footprint levels in the 5' UTRs when an active RNase is present vs the respective control.

In all panels data shown for RNase L activation (+2-5A) was obtained from [25].

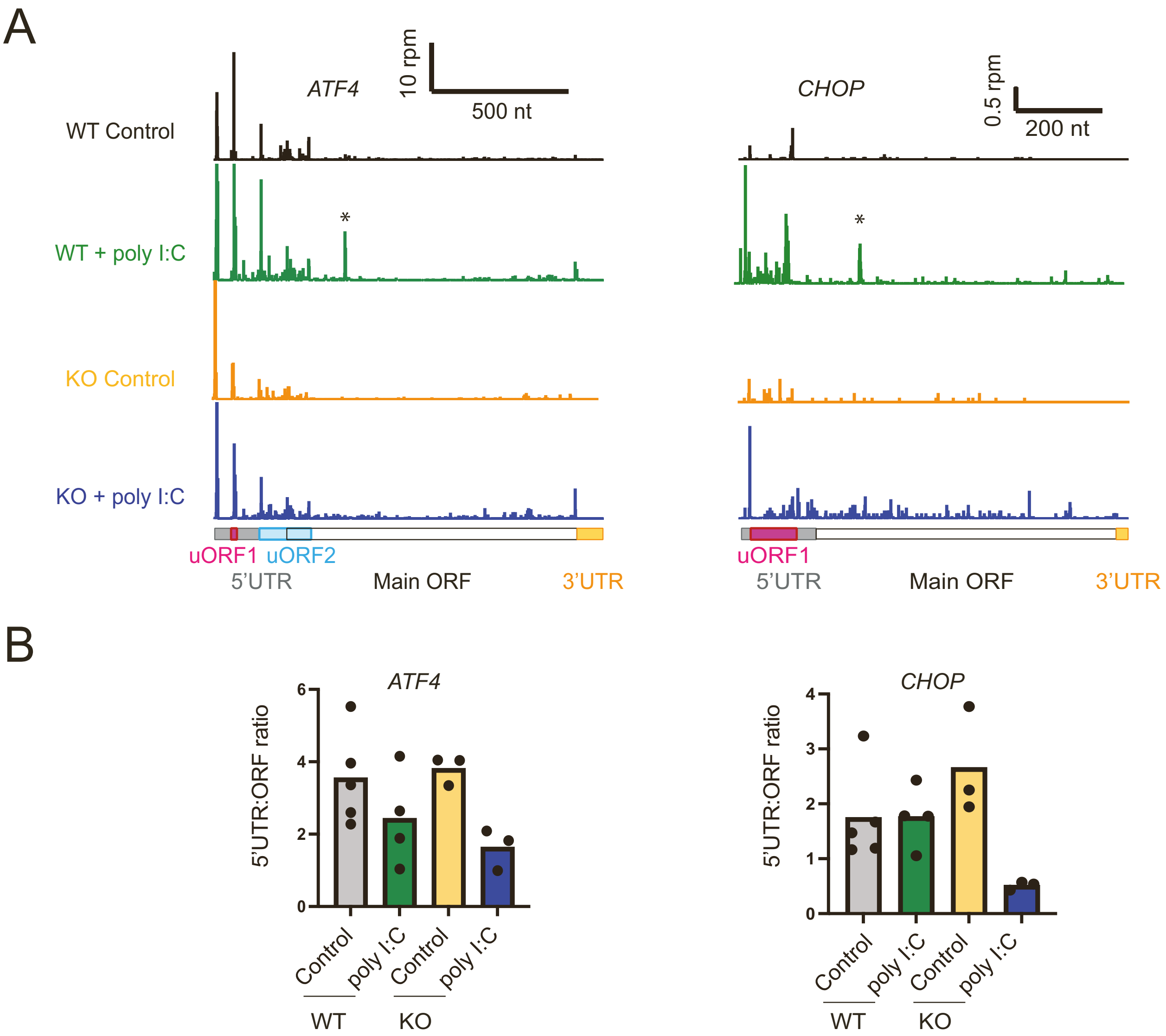

S6 Related to Figure 5. *RNase L modulates elements of the Integrated Stress Response.* **A** Ribosome profiling tracks for gene model of *ATF4* and *CHOP* in WT and *RNASEL* KO cells during 2-5A or poly(I:C) treatment. Data show the poly(I:C) dependent shift toward main ORF vs 5'UTR translation is greater when RNase L is absent. Asterisks show RNase L dependent ribosome profiling peaks in 2-5A and poly(I:C) treated cells that likely correspond to altORF translation initiation events. **B** 5'UTR:main ORF ratios computed from ribosome profiling data in WT and *RNASEL* KO cells during poly(I:C) treatment for *ATF4* and *CHOP* (*n* = 3-5).

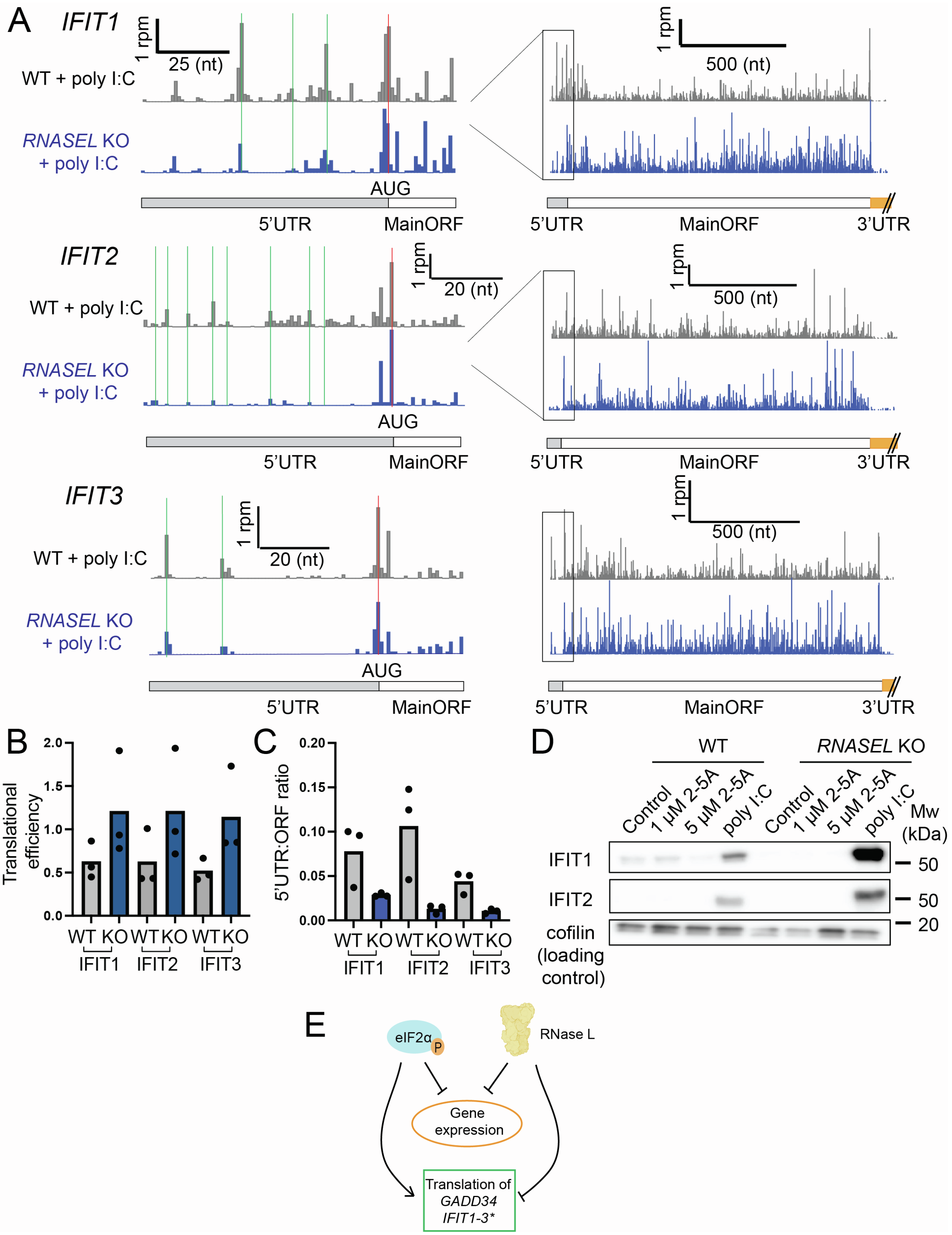

A
IFIT1
1 rpm
25 (nt)
WT + poly I:C
RNASEL KO + poly I:C
AUG
5'UTR
MainORF
1 rpm
500 (nt)
5'UTR
MainORF
3'UTR
IFIT2
1 rpm
20 (nt)
WT + poly I:C
RNASEL KO + poly I:C
AUG
5'UTR
MainORF
1 rpm
500 (nt)
5'UTR
MainORF
3'UTR
IFIT3
1 rpm
20 (nt)
WT + poly I:C
RNASEL KO + poly I:C
AUG
5'UTR
MainORF
1 rpm
500 (nt)
5'UTR
MainORF
3'UTR
B
Translational efficiency
2.0
1.5
1.0
0.5
0.0
WT KO WT KO WT KO
IFIT1 IFIT2 IFIT3
C
5'UTR:ORF ratio
0.20
0.15
0.10
0.05
0.00
WT KO WT KO WT KO
IFIT1 IFIT2 IFIT3
D
WT
RNASEL KO
Control
1 μM 2-5A
5 μM 2-5A
poly I:C
Control
1 μM 2-5A
5 μM 2-5A
poly I:C
Mw (kDa)
IFIT1
50
IFIT2
50
cofilin (loading control)
20
E
eIF2α
P
RNase L
Gene expression
Translation of GADD34 IFIT1-3*

S7 Related to Figure 5. *IFIT mRNAs undergo changes in translation that depend on RNase L.* **A** Ribosome profiling gene tracks for *IFIT1-3* mRNAs. uORFs were identified based on the increase in ribosome density at alternative start codons (CUG, green markings) in poly(I:C) treated WT or *RNASEL* KO cells. Red marking shows the start codon of the main ORF. **B** TEs of *IFIT1-3* mRNAs are decreased in RNase L deficient cells (comparing WT and *RNASEL* KO poly(I:C) treated cells) consistently across replicates (n = 3). **C** *IFIT1-3* mRNAs were found to have less 5'UTR relative to main ORF translation in poly(I:C) treated *RNASEL* KO (abbreviated to KO) cells as compared to WT cells (n = 3). **D** Western blot for *IFIT1* and *IFIT2* shows increased protein levels in poly(I:C) treated RNase L KO cells, suggesting control of TE or RNA degradation reduce levels in WT cells (n = 3). **E** Schematics of how eIF2α phosphorylation and activation of RNase L affects gene expression in the cell. While both mechanisms lead to reduced gene expression (less translation when eIF2α is phosphorylated and less overall mRNA when RNase L is activated), eIF2α phosphorylation increases translation of *GADD34* but RNase L reduces its translation. Similarly, RNase L reduces *IFIT* mRNA translation. It may also be increased via uORF regulation under conditions of eIF2α phosphorylation (marked by asterisk since further confirmation is needed).

Table S1. Results tables from DESeq2 analysis for RNA-seq (WT and *RNASEL* KO, +/- 2-5A, +/- poly(I:C), BSA vs RNase A electroporated). Related to Figure 1-3.
Table S2. Results tables from DESeq2 analysis for ribosome profiling (WT and *RNASEL* KO, +/-2-5A, +/- poly(I:C)). Related to Figure 5.
Table S3. List of genes that are upregulated by JNK/p38 in WT 2-5A treated cells and interferon response in WT poly(I:C) treated cells. This list was used to create violin plots in Figures 2D and 3D. In addition, a longer list of all genes related to JNK/p38 or interferon are given, as derived from the Harmonizome and Hallmark datasets, respectively (see Methods), used in Figures 2C and 3C. Related to STAR Methods.
